# Analysis of E-mental health research: mapping the relationship between information technology and mental healthcare

**DOI:** 10.1186/s12888-022-03713-9

**Published:** 2022-01-25

**Authors:** Tatsawan Timakum, Qing Xie, Min Song

**Affiliations:** 1 Department of Information Sciences, Chiang Mai Rajabhat University, Chiang Mai, Thailand; 2 School of Management, Shenzhen Polytechnic, Shenzhen, Guangdong China; 3 Department of Library and Information Science, Yonsei University, 50 Yonsei-ro, Seodaemun-gu, Seoul, 120-749 Republic of Korea

**Keywords:** E-mental health, E-health, E-therapies, Mental health, Text mining, Research trends

## Abstract

**Background:**

E-mental healthcare is the convergence of digital technologies with mental health services. It has been developed to fill a gap in healthcare for people who need mental wellbeing support that may not otherwise receive psychological treatment. With an increasing number of e-mental healthcare and research, this study aimed to investigate the trends of an e-mental health research field that integrates interdisciplinary fields and to examine the information technologies is being used in mental healthcare. To achieve the research objectives, bibliometric analysis, information extraction, and network analysis were applied to analyze e-mental health research data.

**Methods:**

E-mental health research data were obtained from 3663 bibliographic records from the Web of Science (WoS) and 3172 full-text articles from PubMed Central (PMC). The text mining techniques used for this study included bibliometric analysis, information extraction, and visualization.

**Results:**

The e-mental health research topic trends primarily involved e-health care services and medical informatics research. The clusters of research comprised 16 clusters, which refer to mental sickness, e-health, diseases, information technology (IT), and self-management. The information extraction analysis revealed a triple relation with IT and biomedical domains. Betweenness centrality was used as a measure of network graph centrality, based on the shortest path to rank the important entities and triple relation; nodes with higher betweenness centrality had greater control over the network because more information passes through that node. The IT entity-relations of “mobile” had the highest score at 0.043466. The top pairs were related to depression, mobile health, and text message.

**Conclusions:**

E-mental related publications were associated with various research fields, such as nursing, psychology, medical informatics, computer science, telecommunication, and healthcare innovation. We found that trends in e-mental health research are continually rising. These trends were related to the internet of things (IoT) and mobile applications (Apps), which were applied for mental healthcare services. Moreover, producing AI and machine learning for e-mental healthcare were being studied. This work supports the appropriate approaches and methods of e-mental health research that can help the researcher to identify important themes and choose the best fit with their own survey work.

**Supplementary Information:**

The online version contains supplementary material available at 10.1186/s12888-022-03713-9.

## Background

The demand for mental health services has been growing globally in recent years. Mental health conditions arise as a result of genetic factors, life circumstances, and other illnesses. This is especially prevalent in people with chronic diseases such as cancer, diabetes, and high blood pressure [1]. Lower social and economic barriers have led to more individuals coming forward for help with mental illness [2]. However, mental health service resources have not been progressing to meet the growing demand. This has led to pressure on service capacity [3, 4], resulting in longer waiting times for people in need of support. Additionally, there are extensive gaps between service engagement and treatment, which is reflected in the high number of people who never receive any treatment.

Digital health is an interdisciplinary field that integrates digital technologies with health, healthcare, and society to enhance the efficiency of healthcare delivery and make medicines more personalized and precise. As part of this, electronic mental (e-mental) health care has been developed to support mental wellbeing by preventing mental illness and the need for psychological treatment [5]. It is part of a solution to directly help individuals in need by utilizing information and communication technology (ICT) via the internet [6, 7]. E-mental health care includes digital technology-based treatments and new media, such as web-based, mobile phone-based, email, and virtual reality-based interventions, and text messaging services for mental health self-management. Additionally, these services are used to distribute health promotion, screening, prevention, and treatment information [8]. They can improve the health care of patients, provide professional education through e-learning, and contribute to electronic research in mental health care [9], cognitive behavioral therapy (CBT) [10], mental health diagnosis, and treat illness-related mental health conditions [11, 12]. Thus, e-mental health care refers to the use of technology and the internet to deliver mental health information and services [13]. This concept covers the range of e-health or digital health services, which are derived from the evolution of technologies like smartphones and mobile devices [14].

Additionally, e-mental health systems can collect individual data to detect mental health symptoms and develop personalized programs that overcome the barriers to seeking help [15]. Tailored treatments may include supportive feedback, CBT, psychoeducation, and acceptance commitment therapy [5]. In recent years, e-mental health treatment programs have incorporated various applications and technologies for smartphone or tablet users that are available to the public. These can be found via apps such as the NHS applications library, which is provided by NHS in England (https://www.nhs.uk/apps-library/category/mental-health/). These applications are assessed against a range of NHS standards. Moreover, there are several mental health services apps for purchasing, such as Moodfit (https://www.getmoodfit.com/), MoodMission (https://moodmission.com/), and Talkspace (https://www.talkspace.com/). Most of these apps allow users to access their features anywhere and anytime. They offer a way to stay engaged, such as feelings trackers and daily reminders. Therefore, the use of IT can assist medical professionals in screening, assessing, monitoring, and delivering treatment interventions as well as supporting the patients with access to mental health care [16].

E-mental health has been studied across various sciences, such as the medicine, psychology, information science, media, and technology related fields. The field has gained a steady increase in interest among researchers. One study has investigated different types of e-mental health self-management interventions [8]. Other studies have analyzed the effectiveness of technology-based self-help therapies and behavioral interventions for mental health problems [17, 18], such as computerized treatment for addictive disorders [5], online treatment for depression [10], and web-based interventions for alcoholism [19]. Another study has analyzed the benefits and negative impact of social network services on the internet [20]. These studies have shown some potential benefits to e-mental health, which have developed with the dynamics of new scientific discipline.

However, in this research field, the analysis of the trends and the IT elements in e-mental health has not been performed. Therefore, to facilitate future research and practice in this field, it is essential to comprehend the emerging subjects and knowledge structures in e-mental health such as medical informatics and artificial intelligence (AI). Moreover, an understanding of the relationship between the domains of biomedical and IT is vital for mental health interventions that can enable a better understanding of the association between diseases and treatments. In this study, mental health is defined as a mental disease and/or a set of mental conditions or symptoms which could correspond with physical sickness. Therefore, the associated diseases and symptoms also should be examined.

Consequently, this study aimed to identify the key attributes of e-mental health by examining bibliographic information and full-text papers. We sought to detect research clusters, entities, and their connections between the biomedical (diseases, symptoms, and treatments) and IT domains. In particular, the present study was designed to answer, 1) What are the research clusters and trends in e-mental health? 2) What kind of IT is being used in e-mental health? and 3) What are the common diseases connected with mental health conditions, and how can IT be used for treatment?

## Methods

### Data collection

The data used for visualizing the research clusters and time series were downloaded under the research topic of e-mental health from Web of Science (WoS) in bibliographic records. The data for information extraction was obtained from full-text papers from PubMed Central (PMC) databases. Both datasets were retrieved on December 16, 2019.

We included “ehealth” *OR* “mhealth” in the search words because they are widely used in the electronic health domain; therefore, we included these words to collect all data related to electronic mental health. However, to obtain the appropriate data for our study, we limited our searching criteria by using *AND* “mental” *OR* “emental.”

The WoS data were collected by limiting the topic search to “eHealth” *OR* “mHealth” *AND* “mental” *OR* “eMental,” selecting English as the language, and limiting the document types to “Article *OR* Review *OR* Meeting Abstract *OR* Editorial Material *OR* Proceeding paper.” We selected the timespan from 1990 to 2019, and the collection indexes included were the Science Citation Index Expanded, Social Science Citation Index, Arts and Humanities Citation Index, and Emerging Sources Citation Index. The WoS dataset included 3663 records (Bibliographic record).

For the PMC data, the keywords were limited to “emental” [Body - All Words] *OR* “ehealth” [Body - All Words] *OR* “emental health” [Body - All Words] *OR* “mhealth” [Body - All Words] *AND* “mental” [Body - All Words]. The full-text results included 3172 records (full-text XML format).

### Data processing

The research was designed to analyze the clusters and trends of e-mental health research and extract the entities of biomedical (diseases, symptoms, and treatments) and IT domains. Further, we investigated the association between those entities. We used visualization tools to present the research clusters and entity co-occurrence. The proposed approach of this study is explained, as follows.

First, bibliometric analysis was performed. This is a quantitative statistical analysis approach that is used in the study of scientific citations in academic communication systems [21]. This approach explores the research topics and trends, then evaluates the productivity of authors and cooperative networks in specific fields [22]. Using this method, the collaboration patterns between authors, institutions, and publishers are captured to better understand global trends and discover research frontiers [23]. Mapping science helps to depict the knowledge structure in scientific networks and discover growing discipline areas. This study used the WoS data to obtain a comprehensive understanding of the clusters of e-mental health research. In addition, we created a time series and analyzed the trends in the data. The WoS analysis tool (Clarivate Analytics) was used to observe the research trends. Citespace.5.5.R2 [24] was used to examine and visualize the clusters of e-mental health and time-series of them. The period from 1990 to 2019 was selected for the study. The bibliography data from WoS was analyzed based on collaboration relationships using a reference and cited author. The sources were represented by the title, abstract, author keywords (DE), keywords plus (ID), and node type was shown by the term and keyword. The top 50 most cited or occurring items were selected for visualization from each year slice. This process was applied to answer research question (RQ) 1.

Second, information extraction (IE) was performed. This is a text mining technique to pull useful information from text documents. It is a part of Natural Language Processing (NLP) that is used for tasks, including Named Entity Recognition (NER) and Relation Extraction (RE) [25]. The NER system recognizes a named entity that occurs in the text, such as the name of a person, organization, or specific category. The RE system detects and classifies the relationship between entities in the text. The shared functions in the IE approach have significantly contributed to identifying patterns of knowledge in health and biomedicine. We applied this method to identify the biomedical and IT entities and their connections in the full-text papers from PMC using the PKDE4J 2.0 knowledge discovery tool [26]. This system integrates dictionary-based entity extraction and rule-based relation extraction. To identify the entities of e-mental Health, four types (dictionaries) were selected, including “Information Technology”, “Disease”, “Symptom”, and “Treatment.” These were used as inputs for the tool. In the treatment dictionary, we included medical procedure terms that are used to measure, diagnose, monitor, and treat a problem or disease. Therefore, they were classified into the same category. To use these dictionaries, we considered a description of self-management [27], which relates to the management of symptoms, treatments, and physical and mental conditions (diseases). The biomedical dictionary was already included in the PKDE4J biomedical entity and relation extraction package [26]. The corpus used in PKDE4J was compiled from clinical healthcare terminologies such as the National Library of Medicine’s controlled vocabulary thesaurus (MeSH) [28], the National Center for Biotechnology Information (NCBI) disease corpus [29], and clinical terms [30]. Bio entities with three entity types (Disease, Symptom, and Treatment) were constructed from these collections.

The IT lexicon was collected from IT resources, such as the TechTerms [31] and Computer Hope [32], which provide support related to the internet, software, artificial intelligence, cell phones, internet, smartphones, sound, video, and IT security. A summary of the dictionary data is displayed in Table 1. In addition, the system incorporated biomedical verbs, which were extracted from the unified Medical Language System [33].

**Table 1:** Dictionaries for entity analysis

Dictionary	Word count	Word examples
Information Technologies	39,447	videoconferencing, image, visual information, mobile internet technologies, mobile Health, virtual reality, smartphone, voicemail, texting, short message
Diseases	71,234	breast cancer, acute asthma, eating disorders, severe asthma, bipolar, unipolar depression, manic depression, major depression, social phobia
Symptoms	387	depression, tightness, phobia, photophobia, nausea, migraine, inflammation, fatigue. Pain, scared, suicide attempt
Treatments	7800	radiation, chemotherapy, surgery, behavior assessment, medical photography, breast pneumocystogram, treatment, expression, suicide prevention

Finally, we performed network analysis and visualization to present the connections of each entity type. Network analysis is a method derived from network theory. It emerged from computer science to illustrate the influence of social networks [34]. It allows researchers to describe relationships between entities [35]. Social network analysis has been applied in several fields, including the science of citation, which are presented in graph theory [36]. Concept graphs consist of sets of nodes and edges that are used to represent text documents [37]. These create visual links and measure the impact of each node based on their pairs in a network. This technique determines which terms are used as a bridge in a network. Therefore, it is necessary to calculate metrics such as the weighted degree of nodes [38] and betweenness centrality [39], which are crucial to analyzing the co-occurrence network. This analysis was performed to answer RQ 2.

Data pre-processing included abbreviation resolution, tokenization, sentence splitting, POS tagging, lemmatization, and string normalization [26]. These techniques were applied to perform a sentence-level analysis of both datasets. They were then processed through Named Entity Recognition (NER), which is a dictionary-based approach. The NER consists of N-gram matching, approximate string match, regex NER, candidate entity filtering, and labeling. Lastly, data were delivered to post-preprocessing and rule generation to be assigned an entity name and entity type.

**Fig. 1:**
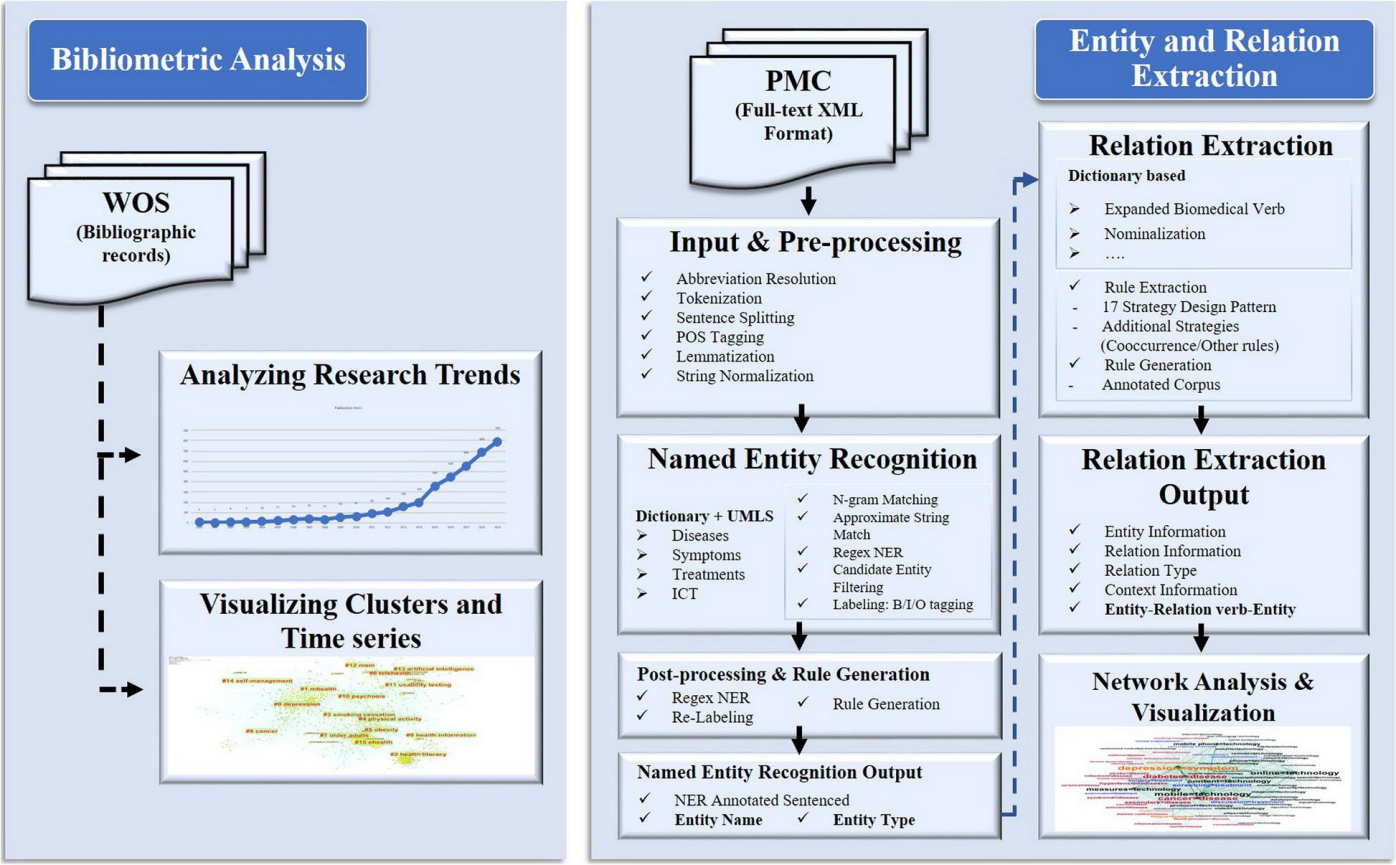
Research framework

After extracting and receiving the entity and relation results from 3172 full-text papers, the relationships were used to construct two different networks of entities to be analyzed. First, a network of four entity types and their connections were examined to provide an overview and visualize the prominent pairs in the PMC dataset. Second, a graph of common diseases was created to illustrate the connections between the IT, symptoms, and treatments entities. The nodes (entities) and edges (relations/connections) were evaluated based on betweenness centrality and weight degree. Later, Gephi 0.9.2 [40] was used to visualize those networks. An overview of all data processing is shown in Fig. 1.

## Results

### E-mental Health Research trends

**Fig. 2:**
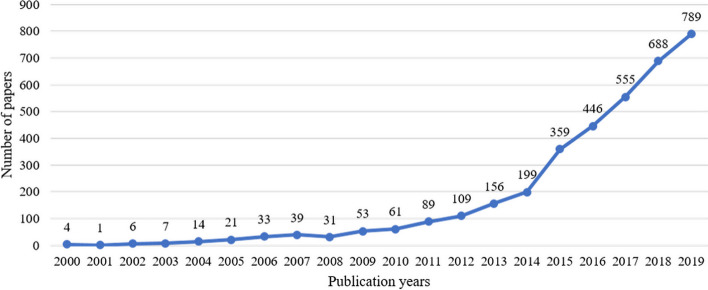
Number of published papers related to e-mental health between 2000 and 2019, indexed by Clarivate Analytics tools

The number of studies in e-mental health research has been increasing steadily (Fig. 2). In 2015, there was a significant change in the number of publications related to e-mental health, rising to 359 records that year and peaking at 789 records in 2019.

Based on the WoS data analysis, the first publication related to e-health research, indexed by the Clarivate Analytics tools, was published in 2000. This was a study on electronic patient records using an internet-based approach [41]. In the same year, a study on the ethics of e-health was published [42]. In 2006, e-health research regarding e-health literacy was prominent. The most cited research paper assessed e-health literacy skills for consumer health [43]. This upward trend in e-health research continued and reached the highest number of studies in 2015. Research included topics such as internet resources for health care [44], mobile apps [45], mobile phone sensors [46], and web 2.0 [47]. One study focused on treatment delivery via mobile apps for bipolar disorder [48]. Since 2019, telehealth services, especially mobile and smartphone applications for mental health and health monitoring, have been well-established in this research field. A telerehabilitation study [49] was the most cited. These results reflect the growing research trends in e-mental health care.

Research area examination by WoS categories showed that the top-ranking field for published research papers associated with e-mental health was health care sciences services (1366 records), followed by medical informatics (1106 records), and computer science (399 records). Other related areas included public health, psychiatry, nursing, telecommunication, and biomedical social sciences as shown in Table 2.

**Table 2:** Top research areas of e-mental health, as measured by Clarivate Analytics tools

Research Areas	Records
Health care sciences services	1366
Medical informatics	1106
Computer science	399
Public environmental occupational health	361
Psychiatry	294
Psychology	287
General internal medicine	177
Oncology	168
Engineering	142
Nursing	104
Information science library science	92
Telecommunications	82
Biomedical social sciences	65
Cardiovascular system cardiology	62
Research experimental medicine	60
Neurosciences neurology	54
Rehabilitation	53
Social sciences other topics	53
Endocrinology metabolism	50
Education educational research	49

### Mapping the clusters of E-mental Health Research

Bibliographic analysis revealed that the most frequent document type in the co-citation network was articles, with a total of 10,834 items. This was followed by review papers (2211 items), early access articles (1140 items), meeting abstracts (575 items), editorial material (404 items), and reviews of early access (210 items). The network included 1392 nodes and 2784 edges.

**Fig. 3:**
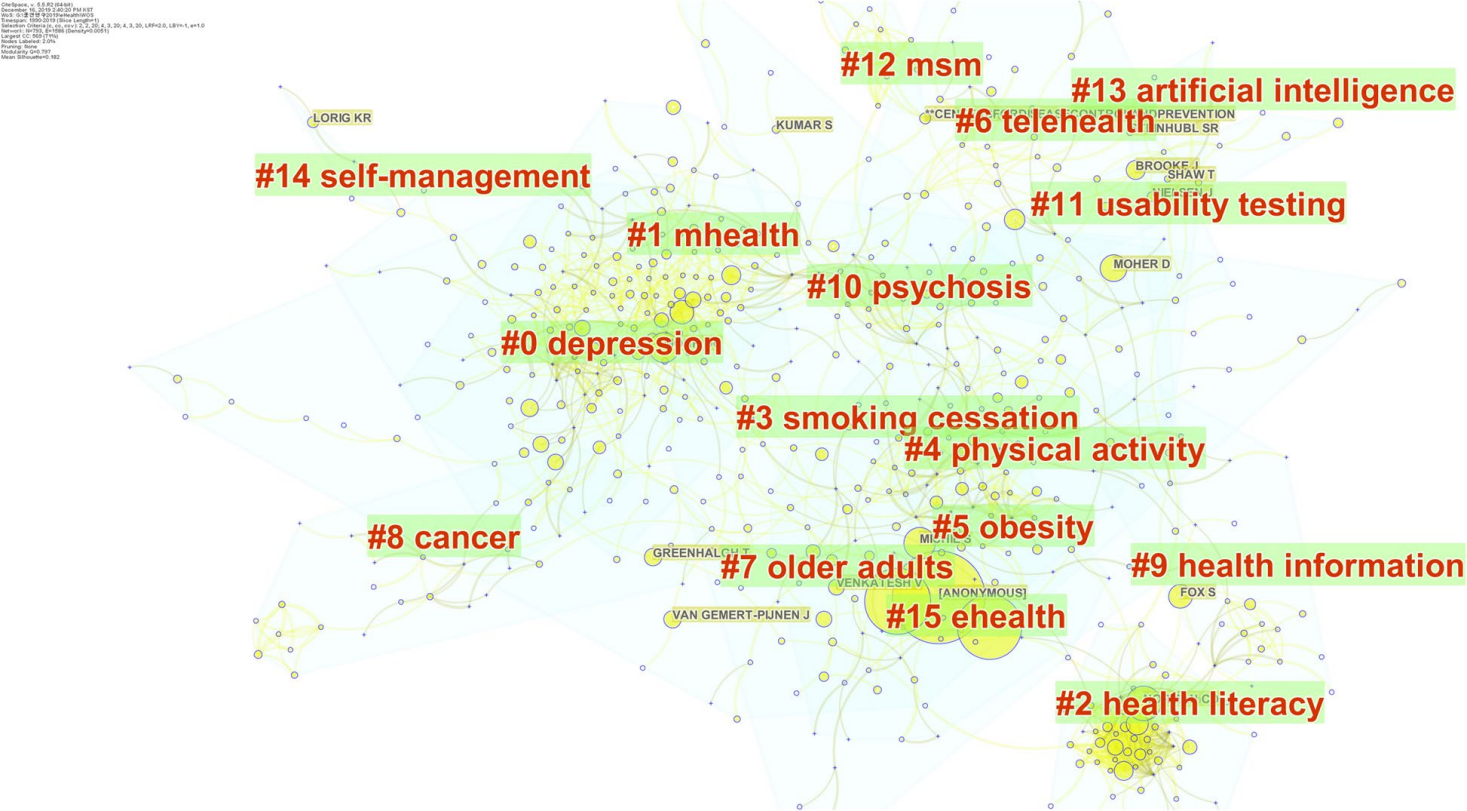
E-mental health care research clusters

The system detected meaningful research clusters from the co-citation network in 16 groups (Fig. 3). The cluster name was determined using the keywords in the corresponding cluster, which is shown in a full result at the DOI link (Additional file 1: Appendix 1). Cluster #0, “depression,” was the largest research cluster with 82 papers. This cluster is associated with mental health, noncommunicable diseases, breast cancer survivors, and cognitive-behavioral. This cluster was followed by cluster #1, “mhealth,” (74 papers), which included papers on mental health, user engagement, alcohol consumption, mobile apps, and e-mental health. Cluster #2, “health literacy,” had 56 papers. This included papers addressing the theory and technique of psychological knowledge evaluation, shown in terms of psychometrics, classical test theory, item response theory, and social support.

The rest of the clusters were #3 “smoking cessation,” #4 “physical activity,” #5 “obesity,” #6 “telehealth,” #7 “older adults,” #8 “cancer,” #9 “health information,” #10 “psychosis,” #11 “usability testing,” #12 “msm,” #13 “artificial intelligence,” #14 “self-management,” and #15 “ehealth.” Some clusters refer to mental disorders and related diseases and symptoms, including smoking cessation, obesity, cancer, and psychosis, which are associated with mental health challenges. Some clusters represent a treatment using IT for self-management, such as the clusters for physical activity, telehealth, health information, self-management, and ehealth. Other clusters represent usability design for the participants, as demonstrated by the cluster for usability testing and artificial intelligence. The most common target demographics were elderly people and men who have sex with men (MSM).

### IT and biomedical entities and relations

In this study, four entity types were considered: IT, treatment, disease, and symptom. The relation extraction process was performed to detect co-occurrences between these entities within a sentence of the data corpus. Table 3 shows the results of the named entity extraction in the collected dataset. The most frequently occurring entity type was IT*,* with 667,291 entities and 2290 unique entity names. This was followed by the treatment (106,519), disease (78,622), and symptom (17,474). The disease entity had the most unique names (2765). By contrast, the symptom entity had only 106 unique names. The top 30 entity names for each entity type are shown in Table 5, along with their influence degree in the created network.

**Table 3:** Entity extraction results

Entity type	Entity counts	Entity names
IT	667,291	2290
Treatment	106,519	787
Disease	78,622	2765
Symptom	17,474	106

After extracting the entities, the system determined the connections between any two entities found in the same sentence and connected them with a relation verb. Table 4 displays the relation extraction results with the total numbers of co-occurrences. The connection between IT and IT was perceived the most frequently (777,788 counts). However, we did not focus on this co-occurrence since the study aimed to investigate the association of IT with diseases, symptoms, and treatments. The co-occurrence of IT and treatments occurred 125,199 times, followed by IT and diseases (96,732), and IT and symptoms (18081).

**Table 4:** Relation extraction results

Entity pairs	Total co-occurrences
Technology | Technology	777,788
Technology | Treatment	125,199
Treatment | Technology	
Technology | Disease	96,732
Disease | Technology	
Technology | Symptom	18,081
Symptom | Technology	

The relation extraction process identifies the co-occurrence of entities at the sentence-level, in which the entity extraction module has extracted two or more entities. The relationship analysis module takes a list of verbs and nominalization words that are used to identify relationships of interest. For example, the Entity 1 *“text messaging”* (IT) connects to Entity 2 *“smoking cessation”* (Disease) with the relation verb *“target,”* which were extracted from the same sentence. This result was detected from the following sentence in the dataset.

*“Thus, SMStext messaging*^(Entity 1)^*might be an appropriate way totarget*^(Relation verb)^*smoking cessation*^(Entity 2)^*in low SES and African American smokers”.*

After we obtained the results of entity and relation extraction, we further employed them in the network analysis to determine the degree of each entity (node) and relation (edge). These are displayed as a graph for easier interpretation.

### E-mental health entities network analysis

The entity and relation extraction results were passed into a graphML formatting process to create a network and were exported to Gephi for visualization. The network was evaluated using betweenness centrality to produce a bigger graph that combines all of the entities, relations, and weighted degrees for a specific network, such as a specific disease with IT. Betweenness centrality for a node represents the degree to which the nodes are mutually connected [50]. Thus, a node with higher betweenness centrality will be more important than other nodes because more information will pass through that node. In other words, the higher weighted entities indicate a higher impact.

### IT and biomedical entities network

**Fig. 4:**
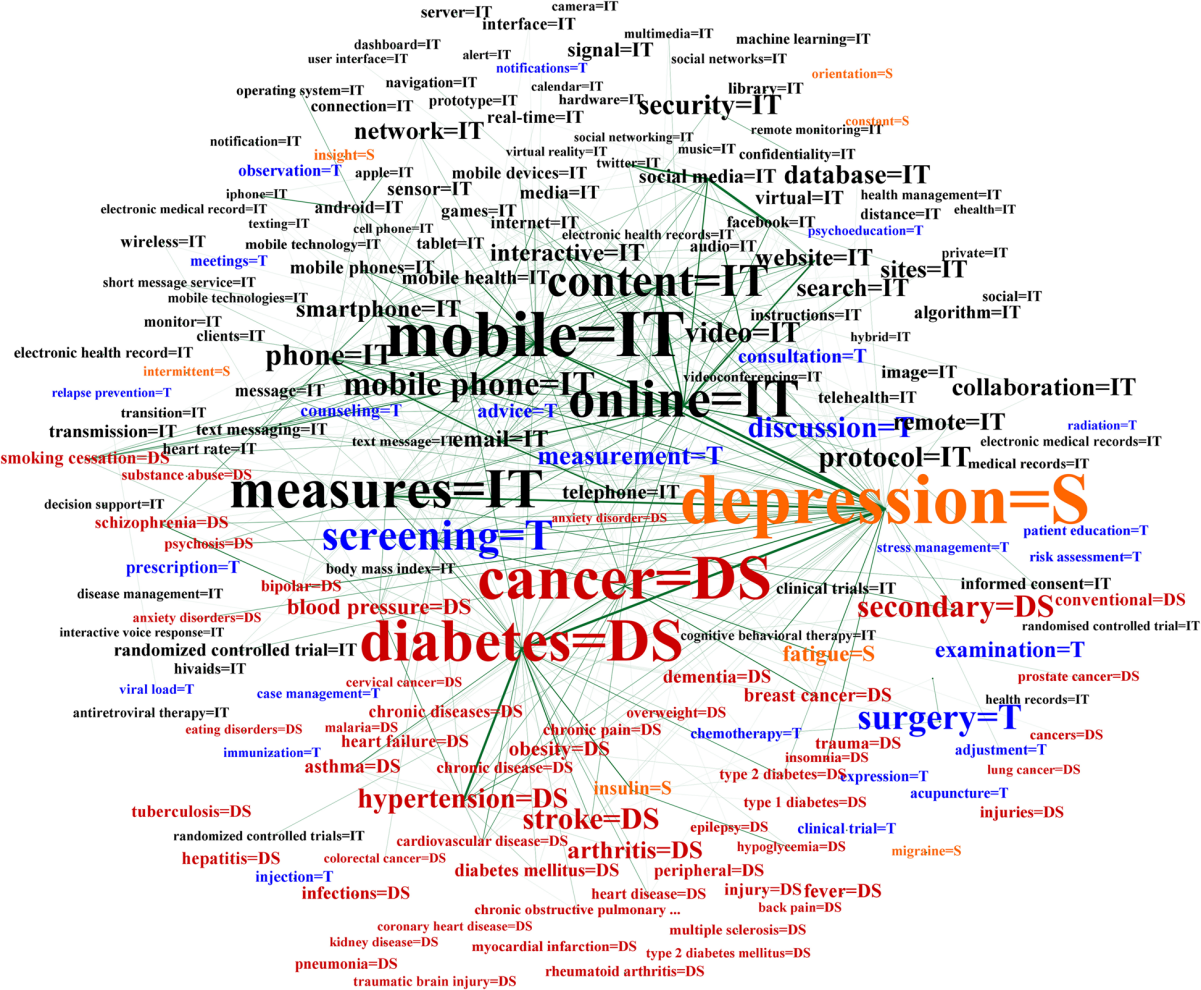
Network of entities and relations in the e-mental health research field

The network was processed according to the shortest path between each entity pair to produce the graph. The network shows the entities and connections of four entity types, including technology (used for IT), disease, symptom, and treatment. The network is an indirect graph that integrates the related research of e-mental health in a total of 7025 nodes and 105,621 edges (Fig. 4). Each node refers to the extracted entities from 3172 full-text papers. The edge indicates the connections between nodes.

**Fig. 5:**
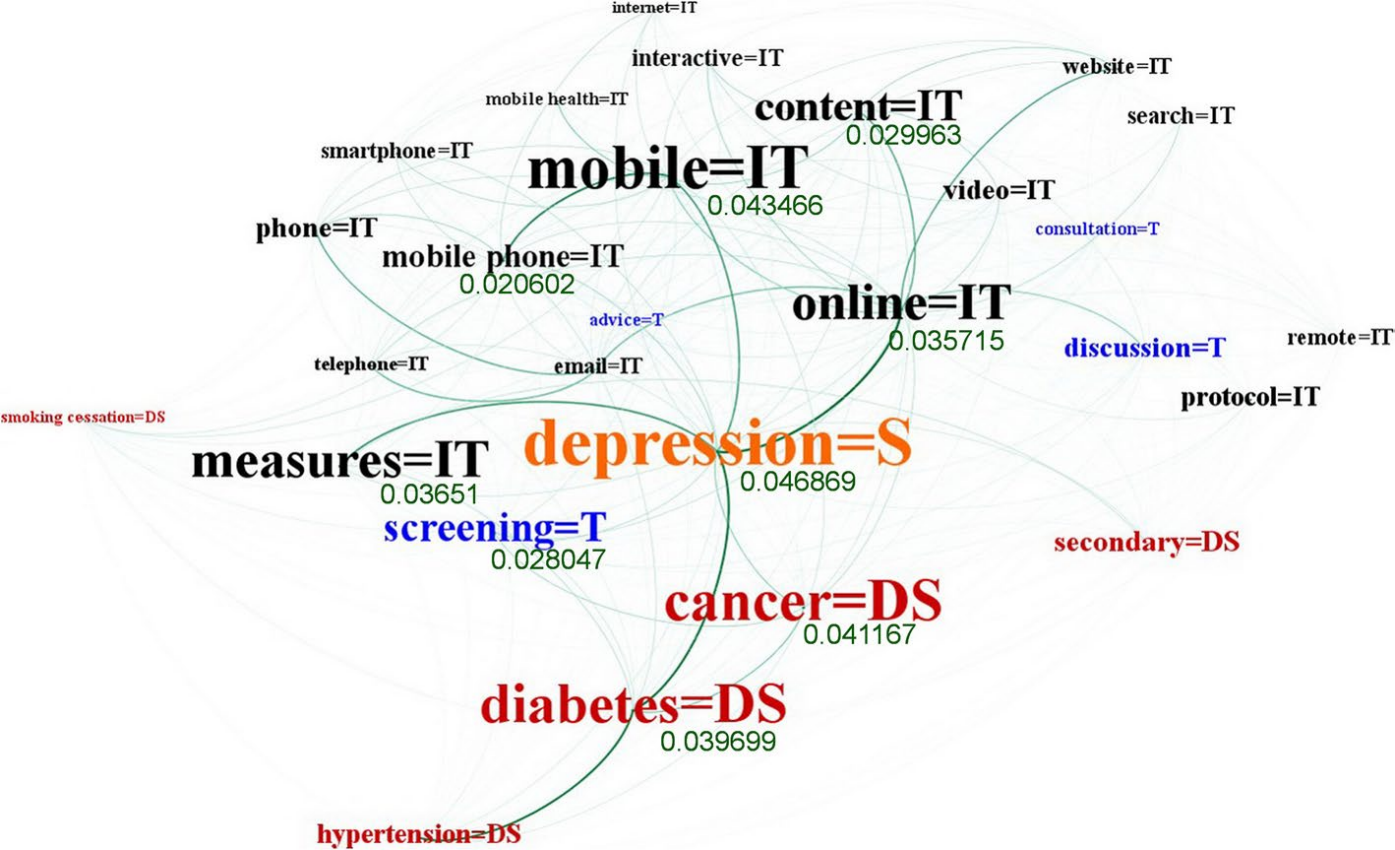
Influent entities connections in the e-mental health network

Among the 7025 nodes and 105,621 edges, the unigram entity “depression” was the biggest node with a betweenness centrality at 0.046869, followed by “mobile” (0.043466), “cancer” (0.041167), and “screening” (0.028047). The “depression” node had a high influence on other nodes, including “cancer,” “diabetes,” “mobile,” “online,” “measures,” “content,” “screening,” and “discussion” (Fig. 5).

As shown in the large network of e-mental health entities and relations (Fig. 4), the results indicate that the technology (IT) nodes strongly occurred with “mobile,” “measure,” “online,” “content,” “video,” “protocol” (communication protocol)*,* and “security.” In addition, the entities of multimedia for self-monitoring and facilitation in therapy were visualized, including “video,” “sites,” “email,” “social media,” “virtual,” “image,” and “text messages”. Smart devices were frequently identified, such as “mobile phones,” “smartphones,” and “sensors.” The operating system entities such as “protocol,” “remote,” “algorithm”, and “android” were discovered in the network.

Figure 5 shows various associations between IT entity nodes and other nodes. Analysis showed that the node “measures” was associated with the IT entity type itself (mobile, online, content, smartphone, and interactive), disease entities (cancer, diabetes, secondary, hypertension, arthritis, stroke, and blood pressure), and with symptom entities (depression, fatigue, and discharge). Moreover, it is linked to treatment nodes, including “screening,” “discussion,” “measurement,” “examination,” and “advice.” Interestingly, the treatment entity type is frequently represented by the entities “screening,” “surgery,” “discussion,” “measurement,” and “examination.” As displayed in Fig. 5, “screening” co-occurred with other nodes (diseases, symptoms, and technology), such as cancer, depression, and measures, respectively. In terms of supporting treatment, IT was most frequently used as a tool for “screening” to assist first-line physicians and identify potential health problems or diseases, such as detecting mild cognitive impairment and diseases at an early stage. The entity “surgery” was also a significant node, which implied that this treatment type was associated with telemedicine in surgery and online counseling for specific diseases. Moreover, the network illustrated that talk therapy was a conventional treatment in the e-mental health research field, as demonstrated by high-frequency entities such as “discussion,” “consultation,” and “advice.”

Following the entities network result, Table 5 presents the top 30 highest ranked nodes based on the betweenness centrality degree are reported for each entity type.

**Table 5:** Top 30 of each entity type ranked by betweenness centrality

Technology *(Betweenness centrality)*	Treatment *(Betweenness centrality)*	Disease *(Betweenness centrality)*	Symptom *(Betweenness centrality)*
Mobile	Screening	Cancer	Depression
*0.043466*	*0.028047*	*0.041167*	*0.046869*
Measures	Surgery	Diabetes	Fatigue
*0.03651*	*0.022244*	*0.039699*	*0.010418*
Online	Discussion	Secondary	Discharge
*0.035715*	*0.017118*	*0.018623*	*0.007438*
Content	Measurement	Stroke	Insulin
*0.029963*	*0.013998*	*0.017603*	*0.007111*
Mobile phone	Examination	Hypertension	Insight
*0.020602*	*0.010859*	*0.015674*	*0.00292*
Phone	Advice	Arthritis	Migraine
*0.017235*	*0.007979*	*0.012551*	*0.001742*
Video	Consultation	Blood pressure	Orientation
*0.016899*	*0.007741*	*0.010385*	*0.00143*
Protocol	Counseling	Obesity	Intermittent
*0.016559*	*0.005387*	*0.008463*	*0.001223*
Security	Prescription	Breast cancer	Constant
*0.015979*	*0.005225*	*0.00732*	*0.000688*
Database	Observation	Fever	Burning
*0.014449*	*0.004164*	*0.007283*	*0.000664*
Search	Injection	Asthma	Necrosis
*0.013197*	*0.003366*	*0.007114*	*0.000598*
Network	Meetings	Dementia	Swelling
*0.01292*	*0.003202*	*0.006455*	*0.000406*
Sites	clinical trial	Diabetes mellitus	Breathlessness
*0.012854*	*0.003072*	*0.006232*	*0.00034*
Collaboration	Expression	Infections	Dysplasia
*0.012822*	*0.002974*	*0.005956*	*0.000298*
Interactive	Chemotherapy	Conventional	chest pain
*0.012583*	*0.002935*	*0.005703*	*0.000286*
Smartphone	Adjustment	Hepatitis	Mucosa
*0.012131*	*0.00276*	*0.00563*	*0.000284*
Website	Acupuncture	Smoking cessation	Scared
*0.012016*	*0.002377*	*0.005504*	*0.000284*
Remote	risk assessment	Chronic diseases	Tender
*0.011993*	*0.001961*	*0.005452*	*0.00028*
Email	patient education	Heart failure	Fracture
*0.011864*	*0.00194*	*0.005305*	*0.000246*
Signal	Notifications	Peripheral	Suicidal thoughts
*0.008645*	*0.001752*	*0.00495*	*0.000239*
Telephone	case management	Injury	Shooting
*0.00823*	*0.001569*	*0.004899*	*0.000188*
mobile health	stress management	Schizophrenia	Epidural
*0.007792*	*0.001488*	*0.004834*	*0.000151*
social media	Immunization	Tuberculosis	Neck pain
*0.007778*	*0.001425*	*0.004755*	*0.000119*
Virtual	viral load	Trauma	Irritable
*0.007588*	*0.001253*	*0.004692*	*0.000099*
Sensor	Psychoeducation	Chronic disease	Intoxication
*0.00729*	*0.001195*	*0.003998*	*0.000092*
Media	Radiation	Injuries	Tingling
*0.006693*	*0.000626*	*0.003996*	*0.000092*
Mobile phones	relapse prevention	Bipolar	Irrelevant
*0.006405*	*0.000563*	*0.003833*	*0.000086*
Algorithm	health assessment	Chronic pain	Hyperactivity
*0.006144*	*0.000539*	*0.003811*	*0.000085*
Transmission	physical therapy	Type 2 diabetes	Tightness
*0.005924*	*0.000528*	*0.003218*	*0.00007*
Randomized controlled trial	Scanning	Heart disease	Phobia
*0.005882*	*0.000519*	*0.003002*	*0.000068*

### Common diseases with IT entities network

In this process, we sought to investigate the top diseases associated with mental health and identify the IT used for intervention. After the common disease entities with high-frequency detection in the dataset were selected from entity extraction results, the relation extraction results were filtered out. Only the top 20 common diseases and their relations remained for the network analysis. In this report, the numbers in parentheses are the frequency of each disease that was detected in the PMC dataset. The top 20 disease entities were “diabetes (5424),” “cancer (4898),” “smoking cessation (1733),” “dementia (1656),” “blood pressure (1508),” “stroke (1269),” “breast cancer (1200),” “obesity (1198),” “hypertension (1132),” “schizophrenia (1088),” “type 2 diabetes (860),” “psychosis (798),” “asthma (739),” “arthritis (714),” “bipolar (697),” “chronic pain (697),” “insomnia (502),” “cardiovascular disease (498),” “heart failure (497),” and “anxiety disorders (479).”

Network analysis showed the prominent IT entities related to e-mental health were “online” (weighted degree of 2478), “measure” (2365), “mobile” (2358), “content” (2242), and “video” (2160) (Table 6). In addition, there were other IT terms associated with the top diseases that were discovered in the e-mental health dataset. These included privacy and security concerns (informed consent, security), user interface design (interactive, utility, interface user satisfaction, limited English proficiency), hardware, and software-related terms. A full list is available via the DOI link (Additional file 2: Appendix 2).

**Table 6:** Top 20 diseases entities and weighted degree of IT entities

Top 20 common diseases	IT entities	Weighted degree
1. Diabetes	online	2460
2. Cancer	measures	2456
3. Blood pressure	mobile	2337
4. Smoking cessation	content	2301
5. Dementia	video	2144
6. Stroke	interactive	2052
7. Hypertension	treatment as usual	1993
8. Schizophrenia	mobile phone	1958
9. Obesity	protocol	1790
10. Breast cancer	phone	1766
11. Asthma	email	1613
12. Psychosis	smartphone	1555
13. Bipolar	website	1491
14. Cardiovascular disease	mobile health	1443
15. Heart failure	randomized controlled trial	1436
16. Chronic pain	internet	1356
17. Type 2 diabetes	informed consent	1329
18. Anxiety disorders	text messaging	1325
19. Arthritis	instructions	1311
20. Insomnia	monitor	1261

### Entity Association in a Specific Disease Network

**Fig. 6:**
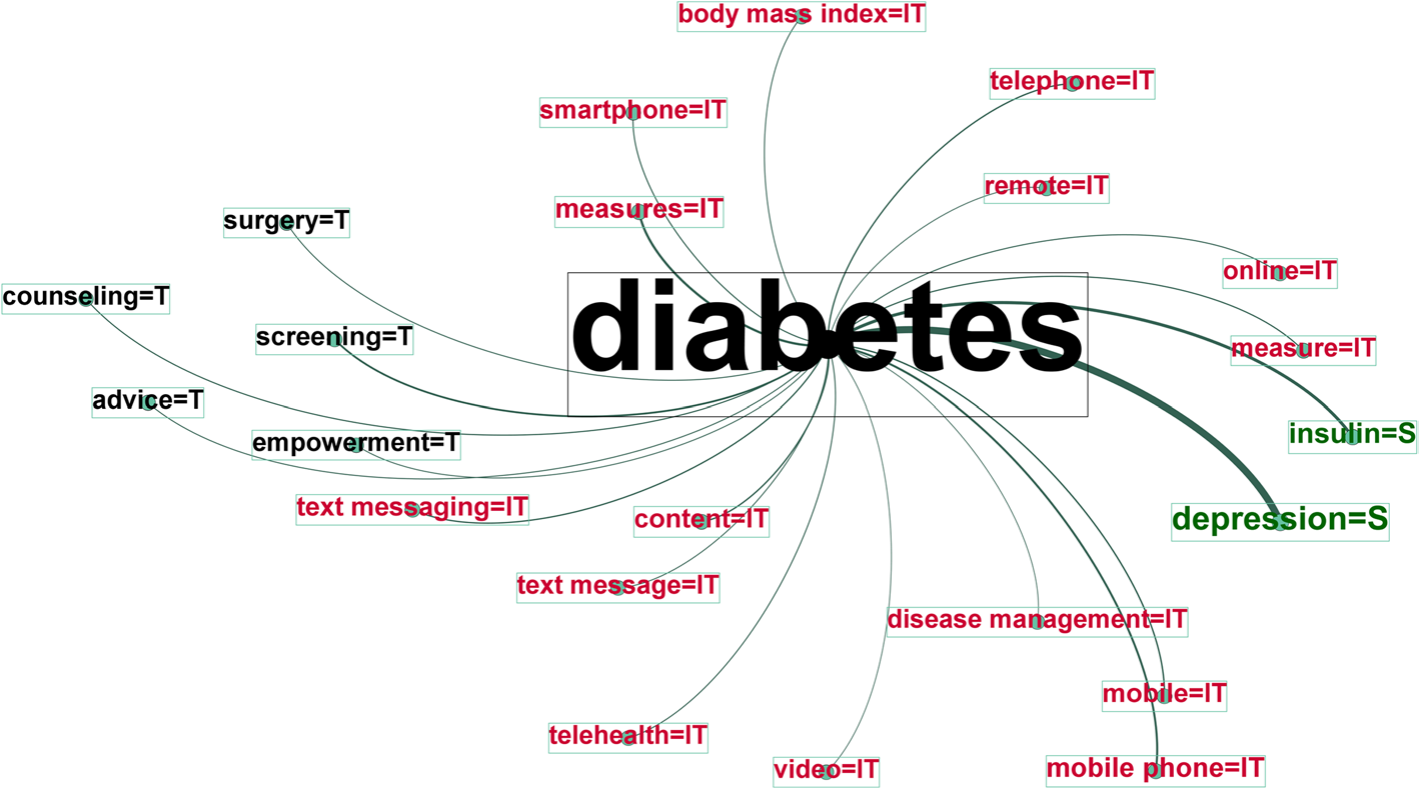
Diabetes entity network

We investigated the entities and relations of the top 20 common diseases in the e-mental health research field. This analysis focused on the relations of four entities (disease, symptom, IT, and treatment) to explore what types of IT were used to treat illnesses and their symptoms. Figure 6 displays the diabetes network, where signs of “depression” (weighted degree score of 219) occurred most frequently for this disease. The significant technology entities were “measures,” “mobile phone,” “content,” “text messaging,” and “smartphone.” The technology applications associated with treatment entities included “screening,” “counseling,” “advice,” “surgery,” and “empowerment.” More information on the results of all 20 diseases is available at the DOI link (Additional file 3: Appendix 3).

**Fig. 7:**
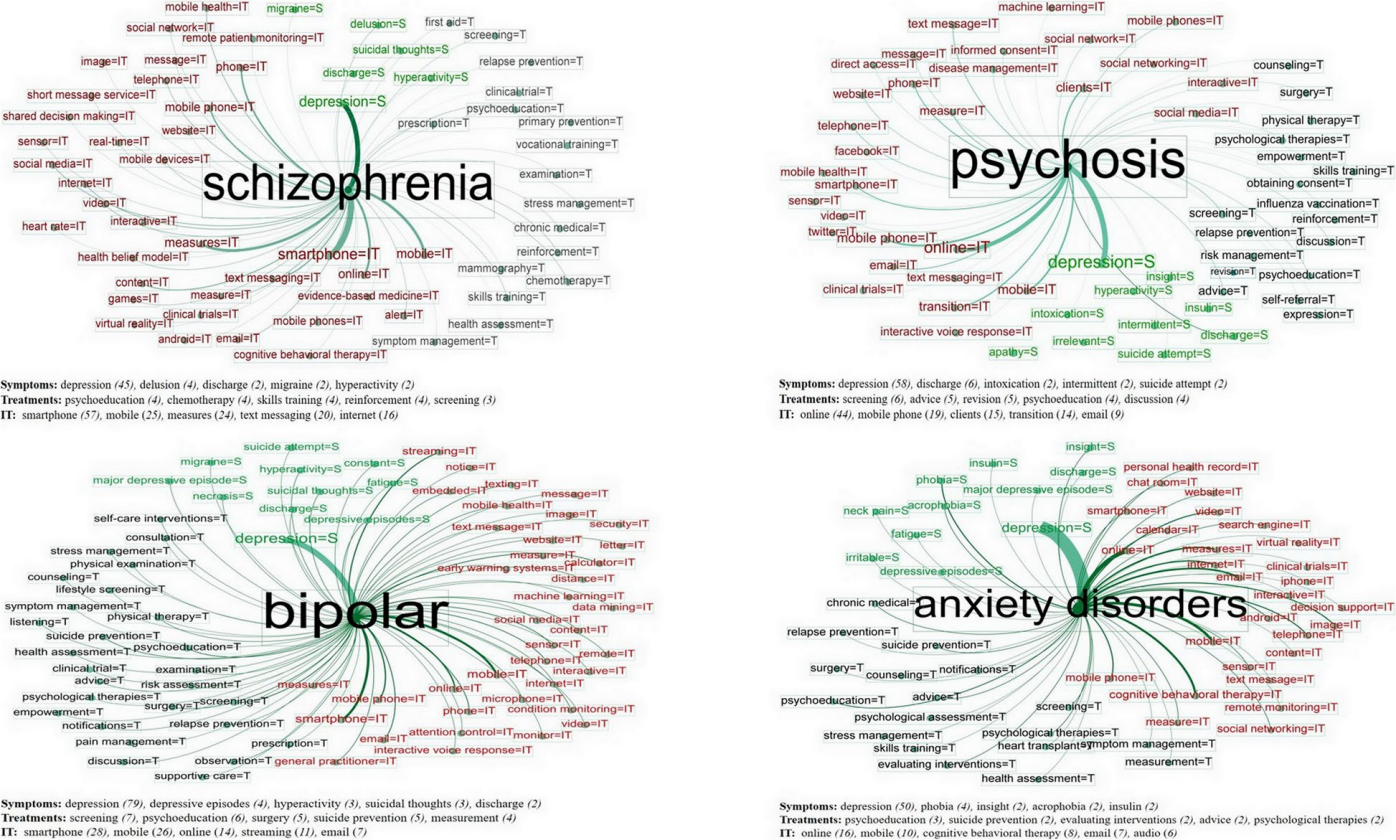
Mental diseases with their associated entities

Figure 7 shows the graphs of four mental illnesses that were detected as the top 20 common diseases, including schizophrenia, psychosis, bipolar, and anxiety disorders. The graphs were adjusted by the weighted degree of entity node for the best view. In these networks, the significant symptoms were “depression,” “suicidal thoughts,” “suicide attempt,” “hyperactivity,” and “discharge.” The prominent treatment entities included “psychoeducation,” and “screening,” and “suicide prevention.” Technology entities were frequently associated, such as “smartphone,” “mobile phone,” and “online.” Additionally, IT media entities were identified, such as text messaging, email, image, video, virtual reality, and social networking. Moreover, the IT for data processing was revealed in these networks (e.g., data mining, machine learning, and streaming). The top five entities for each entity type are shown in each graph.

Of the 20 network graphs of diseases, the treatment entity nodes (including medical procedures) were related to the entity nodes of online counseling (e.g., email, chat, message, and video) for discussion, advice, shared decision making, meetings, and scheduling. Additionally, they were related to patient education, which was shown in the nodes for “skills training” and “psychoeducation.” Other treatment-related nodes identified were “an intervention of cognitive behavior,” “supportive care,” “psychosocial assessment and therapies,” and “clinical trials.” Moreover, specific physical symptoms entity nodes mainly occurred within a specific disease. For example, diabetes and blood pressure entities were linked with “insulin.” The smoking cessation entity was related to “chest pain.”

In addition, the entity and relation networks demonstrated that IT entity nodes were connected with the entities of treatments and medical procedures. These were used for evaluating and diagnosing patients’ conditions and for treatments such as “measuring body mass index,” “insulin therapy,” “glucose tolerance test,” “total cholesterol,” “blood glucose monitoring,” “heart rate,” “diastolic blood pressure,” and “systolic blood pressure” nodes.

Taken together, our data showed that related technologies were applied for physical and psychological interventions and therapies. Furthermore, web-based internet interventions and mobile applications were commonly used, as demonstrated by several of the IT entity nodes.

## Discussion

In this study, there are two dimensions to discuss. First, the analysis of e-mental health research clusters and trends using bibliometrics. Second, the co-occurrences between e-mental health biomedical-related entities and IT entities ranked by weighted degree and betweenness centrality. Mental health in this analysis was considered as a single disease and as symptoms that occurred with physical sickness. Therefore, other biomedical entities were identified to observe the relations between them and to link them with IT entities in this research field.

### What are the research clusters and trends in e-mental health?

In order to overview and understand the scope of the e-mental health research field, we collected WoS bibliographic dataset with the timestamp between 2000 and 2019. We analyzed the trends and observed the related research areas using WoS analysis tools (Clarivate Analytics). We found that interest in this research field is increasing; there was significant progress in 2015, with many publications that were ranked highly. We investigated the e-mental health research topics between 2015 and 2019 and found that the trends were associated with various research fields, such as nursing, psychology, medical informatics, computer science, telecommunication, and healthcare innovation. Furthermore, the research trends at this time were related to the internet of things (IoT) and mobile applications (Apps), which were used in mental healthcare services. Our data showed that in the era of digital behavior, the smart home research topic played a role in contributing a mental health intervention.

Smartphone-based mental health interventions have been studied for the screening, monitoring, diagnosis, and reducing symptoms of depression and anxiety. Mobile health apps were designed for digital self-help interventions that allowed patients to interact with providers remotely and for physicians to deliver therapy. In 2019, we found that research related to artificial intelligence (AI), such as brain-computer interfaces, was more prevalent in this research field.

We further applied the co-citation network analysis to explore the significant e-mental health research clusters that had developed in this research area between 2015 and 2019. The finding demonstrated that 10,834 e-mental related papers could be classified into 16 clusters. The biggest cluster was depression, which is a mental symptom and disease. Other signs and sicknesses were discovered, including smoking cessation, obesity, cancer, and psychosis. These are linked to mental illness; quitting smoking is associated with an increased risk of depression [51, 52]. Moreover, cluster-related health information technology was established in this research area. This comprised of mhealth, health literacy, telehealth, health information, and self-management. In addition, the visualization showed a cluster-related application design in the clusters of usability testing and artificial intelligence. We hypothesized that other clusters in this finding were correlated to a target demographic for e-mental health, such as the older adults and MSM clusters.

Our data analysis enabled an overview of the hidden e-mental health research clusters. From these, we could predict the research trends from the top terms and highest citation year. When this research field was first being established (2008–2010), it was associated with cancer survivors and older adults. This indicates that the first intention of e-mental health research was to focus on the use of IT for self-management and the mitigation of mental health impacts from physical sickness. In 2012, research tended to focus on digital mental health interventions related to psychological and lifestyle interventions; the biggest cluster was “depression,” which was a top term in mental health and behavioral therapy. This observation was confirmed by the research clusters, “physical activity” and “psychosis,” which obtained high citations in the same year.

Between 2013 and 2015, research topics of health information, health literacy, telemedicine, and mobile apps were studied frequently. These topics aimed to support mental health education and mental condition prevention. Moreover, in 2015, the research topic related to artificial intelligence (AI) for mental health and mental illnesses became more prevalent as a mental health digital solution. This was interpreted by the cluster and top terms of “artificial intelligence,” “deep learning,” and “medical informatics.” Many existing studies utilized AI to treat and reduce the burden of mental sickness [53, 54, 55, 56].

To summarize, e-mental health research has been developed as part of the electronic health field that initially focused on electronic patient records. Following this, e-mental health was designed to deliver the right care solutions and mental health services in a timely and effective way by using the internet and other related technologies. With these aims, the researchers in this field have been seeking the appropriate digital technologies for people who lived with a mental health difficulty to access mental health services. As shown in our results, the advance of technologies such as AI applications in medicine could be developed into virtual therapists. Improvements in smart devices and natural language processing offers the ability to learn and monitor the moods and thoughts of patients and deliver therapy. However, to develop the smart tools for mental health care, the technology itself needs further study in addition to further integration of different fields of expertise. For example, it would be important to integrate the technology with the science of medicine, psychology, computing, machine engineering, informatics, communication technology, media and usability design, social networking, or health information education. Therefore, to improve e-mental health platforms and make them ‘smarter,’ the complexities of several sciences should be considered as part of this interdisciplinary research. In summary, this research field has relative interactions across biological, psychological, social systems, and technology research fields. Therefore, the current challenge is to study the principles of e-mental health and generate the cooperation of researchers from various sciences to improve interdisciplinary studies.

### What kind of IT is being used in e-mental health?

To understand the linking of biomedical and IT domains in e-mental health, the diseases, symptoms, treatments, and IT entities and relations were analyzed. We used an information extraction system that integrated dictionary-based entity extraction and rule-based relation extraction. This process classified and identified the associations between biomedical and IT entities. Our overview shows the common diseases, including cancer and diabetes; IT of mobile, online, measures, and content; and the treatments related to screening, surgery, and discussion.

After extracting the disease entities, we found 2765 unique disease names. These included physical and mental diseases in the dataset, which confirmed that the physical condition was also assessed in studies of e-mental health. When examining the uses of IT, we used the top 20 diseases to visualize the associated IT for e-mental health. Our findings show 283 IT entity names. These were assessed and classified into four major groups, multimedia, information system, programming, and disease management and therapy. Multimedia referred to a type of content. The media used in e-mental health was shown by related entities, such as video, email, website, text message, virtual, games, video conferencing, and clinical trials. Moreover, the devices mentioned in the data corpus were smartphones, monitors, androids, sensors, tablets, mobile devices, and iPhones.

The information systems deliver and support users with the information needed for their activities effectively and efficiently. The related entities in this study included information security, clinical decision support system, information systems, information and communication technologies, health information systems, computerized decision support, and personal health management. Next, programming occurs, which is the development of a set of instructions for a computer to perform a task. Our results included the following nodes related to programming: algorithm, analysis of variance (ANOVA), query, segment, machine learning, least squares, embedded, and data mining.

Disease management and therapy refer to a system that coordinates healthcare interventions and communications. The following IT entities were associated with disease management and therapy: measures, treatment as usual, instructions, disease management, hivaids, body mass index, cognitive behavioral therapy, heart rate, cardiac rehabilitation, remote monitoring, real-time, case management, interactive voice response, diagnostics, clinical practice guidelines, emergency room, behavior change techniques, health-related quality of life (HRQoL), and test of functional health literacy in adults (TOFHLA).

In this study, we showed that the uses of IT in e-mental health care were developed for healthcare providers, people who have mental illnesses, and people who are physically sick who report mental conditions. These were developed for physical treatment, mental screening, and prevention. For example, people who inject themselves with medication, such as people with diabetes who require insulin, are affected by trypanophobia (needle phobia). This requires a collaboration between psychological medicine and diabetes teams [55]. Cigarette smoking can cause social phobia and anxiety disorder [58, 59]. People who live with chronic pain have an increased risk for suicide [60, 61]. IT was also utilized for patients with mental disorders who have a higher risk of physical difficulties. For example, the elderly with a history of bipolar disorder have a significant risk of developing dementia [62]. Additionally, major depressive and bipolar disorder can lead to accelerated atherosclerosis and early cardiovascular disease in adolescence [63]. Some IT devices were used for specific diseases and symptoms, such as high blood pressure, which can affect mood disorders [64, 65]. Further, low blood pressure is associated with suicidal ideation [66].

In summary, we used entities and relations analysis in e-mental health research papers to describe the connection between physical and mental illness and the uses of IT for specific diseases, symptoms, and treatments.

### Limitations

We acknowledge that our study has a limitation relating to the use of a dictionary for entity extraction because the text mining tools integrate dictionary-based terms to automatically tag bio-entities according to their types. In our method, we relied on MeSH and SNOMED terms to develop our dictionaries for the information extraction system. However, some entities could be incorrectly labelled. For example, some terms could be either a disease name or a symptom; “blood pressure” and “smoking cessation” could be labelled as diseases, and “discharge” “insulin,” and “insight” as symptoms. These errors were due to the ambiguous concepts of an entity. Sometimes they occurred because of a lexical error that the system failed to extract the entire entity. For example, quitting smoking can lead to symptoms of nicotine withdrawal, such as depression, anxiety, and irritability. Consequently smoking cessation was identified as a disease. At the same time, depression, anxiety, and irritability could be diseases or symptoms. Therefore, the context of entities should be carefully checked manually to prevent errors. In the process of data mining, manual annotation is the essential task of preselecting a document for training and evaluating new natural language processing algorithms. Therefore, we plan to improve an automatic method for disambiguating terms in future work.

Dictionaries and rules-based NER are the classical methods that linguists use to manually create a specific rule or special dictionaries according to the characteristics of datasets. However, the diversity and ambiguity of named entity representations create significant challenges to the understanding of natural language. Under different cultures, domains, and backgrounds, the denotations of named entities differ. This is the fundamental problem that named entity recognition technology needs to solve. Different granularity of knowledge representation in a large amount of text data can lead to different degrees of confidence and a lack of normative constraint; therefore, various expressions and unclear references of named entities appear. It is necessary to fully understand the context semantics to further elucidate the entity semantics for recognition.

In future work, deep learning will be applied in NER. Transfer learning versus remote supervised learning will be fully utilized to solve the problem of named entity identification in resource-poor areas and reduce the workload of manual annotation.

## Conclusions

This paper utilized bibliometric and information extraction methods to report on the landscape of the e-mental health research field and the use of IT for mental health treatments. The data on the topic of e-mental health was obtained from WoS and PMC. WoS and Citespace were used as tools to identify research trends and for cluster analysis. The PKDE4J tool was utilized for the IT and biomedical information extraction, which combined the entities of diseases, symptoms, and treatments. Following this, the results of the entities and relations compilation were processed via network analysis. This was visualized using the Gephi tool.

The results indicated that e-mental health research has been increasing, and most studies relate to health care sciences services and medical informatics. The research was comprised of 16 clusters, which included e-health, diseases, IT, and self-management. Additionally, entities for IT, diseases, symptoms, and treatments and their connections were illustrated in network graphs. The most frequently occurring entity was IT, which was categorized as a mobile entity. Relation extraction showed that the most frequent entity association was depression paired with cancer, diabetes, mobile, online, measures, and screening. Overall, our data showed that e-mental health research focused on disease-related depression, suicidal thoughts, and suicide attempts. IT was used, primarily via online and mobile devices, to deliver health content, text messaging, and audio for screening, psychoeducation, advice, and suicide prevention.

The clusters and research trend analysis results demonstrate the key disciplines in e-mental health research. This is useful for researchers to understand the knowledge structure, relevant cross-discipline, and topic trends in this field. These data can support the correct approaches and methods for further e-mental health research and to identify important themes that fit best with future work. Moreover, the biomedical and IT entity and relation extraction results are beneficial for physicians, patients, and their proximal family members to understand and optimally treat patients with mental disorders in both physical and psychological therapy modalities using IT. The use of IT supports physicians to deliver psychological services as well as health promotion. At the same time, patients have accessibility and flexibility for self-monitoring integrated into treatment. In addition, healthcare providers and IT developers could use the data in this study to support e-mental health design. Furthermore, the outcomes of the entity and relation extraction could be utilized for disease prevention because they identify the diseases that could potentially cause mental health problems.

We expected that our findings can increase our understanding of e-mental health and related research areas. Moreover, promotes the variety of disciplines, especially in emerging research fields, such as medical informatics and AI, which can facilitate early mental disease detection and enable a better understanding of disease and treatments. In future studies, we seek to explore the impact of the global covid-19 pandemic on e-mental health.

## Supplementary Information

**Additional file 1.**

**Additional file 2.**

**Additional file 3.**

## Data Availability

Appendix 1: E-mental health research knowledge clusters. 10.6084/m9.figshare.16957621.v1 Appendix 2: The top 20 diseases with the IT used in e-mental health (283 entity names). 10.6084/m9.figshare.16958221.v1 Appendix 3: Top 20 Disease entities and relations in e-mental health. 10.6084/m9.figshare.16958263.v1

## Abbreviations

ANOVA: Analysis of variance
E-mental: Electronic mental health care
ICT: Information and communication technology
IE: Information extraction
IoT: Internet of things
IT: Information technology
NLP: Natural language processing
NER: Named entity recognition
RE: Relation extraction
WoS: Web of Science
PMC: PubMed Central
HRQoL: Health-related quality of life
TOFHLA: Test of functional health literacy in adults
Mhealth: Mobile health
MSM: Sex with men
CBT: Cognitive behavioral therapy
TAU: Treatment as usual
RCT: Randomized controlled trial
