# Supplementary material for: Analysis of E-mental health research: mapping the relationship between information technology and mental healthcare
Source: BMC Psychiatry. 2022 Jan 25;22:57. doi: 10.1186/s12888-022-03713-9 (PMC8787445; doi:10.1186/s12888-022-03713-9)
Supplement: Supplementary file 1 — Additional file 1. [file 12888_2022_3713_MOESM1_ESM.docx]

**Appendix 1**

E-mental health research knowledge clusters

| cluster ID | Size | Mean(Year)Top Terms(LSI) | | Top Terms (log-likelihood ratio, p-level) |
| --- | --- | --- | --- | --- |
| **#0 depression** | 82 | 2012 | mental health; noncommunicable diseases; developing countries; behavior change; consort ehealth; reporting standards; breast cancer survivors; commitment therapy; acceptance; mobile phone \| cognitive behavioral therapy; healthcare disparities; minority health; patient compliance; mobile apps; computer game; accessibility; mental health; professional-patient relations; e-mental health | depression (27.61, 1.0E-4); cognitive behavioral therapy (19.64, 1.0E-4); mental health (16.16, 1.0E-4); anxiety (10.99, 0.001); digital health (6.47, 0.05); stress (6.33, 0.05); treatment (6.33, 0.05); children (6.33, 0.05); meta-analysis (5.74, 0.05); adolescents (5.73, 0.05); physical activity (5.45, 0.05); text messaging (5.29, 0.05); university student (4.67, 0.05); major depressive disorder (4.67, 0.05); web based acceptance (4.67, 0.05); chronic care (4.67, 0.05); brief intervention (4.67, 0.05); effectiveness (4.67, 0.05); heart rate variability (4.67, 0.05); body dissatisfaction (4.67, 0.05); help-seeking behavior (4.67, 0.05); blended (4.67, 0.05); phq-9 (4.67, 0.05); computer-assisted therapy (4.67, 0.05); attitudes (4.67, 0.05); sexual assault (4.67, 0.05); lmics (4.67, 0.05); quality of health care (4.67, 0.05); survey and questionnaires (4.67, 0.05); routine outcome monitoring (4.67, 0.05); web based intervention (4.67, 0.05); commitment therapy (4.67, 0.05); psychologists (4.67, 0.05); emerging technology (4.67, 0.05); pilot study (4.67, 0.05); world wide web (4.67, 0.05); utaut (4.67, 0.05); transients and migrants (4.67, 0.05); health plan implementation (4.67, 0.05); parenting (4.67, 0.05); young adults (4.67, 0.05); group therapy (4.67, 0.05); programs (4.67, 0.05); feasibility study (4.67, 0.05); healthcare disparities (4.67, 0.05); minority health (4.67, 0.05); professional-patient relations (4.67, 0.05); micro-intervention (4.67, 0.05); chronic fatigue syndrome (4.67, 0.05); smartphone app (4.22, 0.05); mental disorders (4.22, 0.05); online (4.22, 0.05); digital technology (3.24, 0.1); usability (3.24, 0.1); mindfulness (3.24, 0.1); communication (3.2, 0.1); adherence (3.16, 0.1); social support (2.79, 0.1); cost-effectiveness (2.53, 0.5); randomized controlled trial (2.53, 0.5); mhealth (2.37, 0.5); mobile apps (2.37, 0.5); education (2.18, 0.5); health information (2.18, 0.5); digital divide (2.18, 0.5); literacy (2.18, 0.5); task shifting (2.11, 0.5); persona (2.11, 0.5); self-help intervention (2.11, 0.5); smartphones (2.11, 0.5); longitudinal studies (2.11, 0.5); primary health care (2.11, 0.5); patient preference (2.11, 0.5); mental healthcare (2.11, 0.5); user-centred design (2.11, 0.5); fear of recurrence (2.11, 0.5); community health worker (2.11, 0.5); self-help (2.11, 0.5); prevalence (2.11, 0.5); nonspecialist health worker (2.11, 0.5); depressive symptoms (2.11, 0.5); cultural competency (2.11, 0.5); accessibility (2.11, 0.5); perceived competence (2.11, 0.5); acceptance and commitment therapy (act) (2.11, 0.5); measure (2.11, 0.5); latinos (2.11, 0.5); patient compliance (2.11, 0.5); perceived information quality (2.11, 0.5); expert study (2.11, 0.5); stress management (2.11, 0.5); emental health (2.11, 0.5); distance counseling (2.11, 0.5); barriers (2.11, 0.5); applied psychology (2.11, 0.5); computerized (2.11, 0.5); patient satisfaction (2.11, 0.5); interview (2.11, 0.5); ethnic groups (2.11, 0.5); blended therapy (2.11, 0.5) |
| **#1 mhealth** | 74 | 2015 | mental health; user engagement; hazardous alcohol use; mobile apps; computer game; digital tools; rheumatology; young adult; e-mental health; noncommunicable diseases \| mobile health; qualitative research; cognitive behavioral therapy; computer game; mobile interventions; serious mental illness; hazardous alcohol use; mobile apps; digital tools; mental health | mhealth (29.42, 1.0E-4); mental health (22.67, 1.0E-4); mobile health (13.23, 0.001); internet (10.97, 0.001); ehealth (9.93, 0.005); schizophrenia (9.4, 0.005); early intervention (9.15, 0.005); mobile apps (8.1, 0.005); smartphone (7.76, 0.01); psychosis (6.14, 0.05); psychiatry (6.05, 0.05); health literacy (5.54, 0.05); trauma (4.57, 0.05); app store (4.57, 0.05); self-management program (4.57, 0.05); coping (4.57, 0.05); users (4.57, 0.05); digital phenotyping (4.57, 0.05); youth mental health (4.57, 0.05); schizotypal (4.57, 0.05); hazardous alcohol use (4.57, 0.05); bipolar disorder (4.57, 0.05); apps (4.57, 0.05); psychological intervention (4.57, 0.05); focus group (4.57, 0.05); technology platform (4.57, 0.05); social representations (4.57, 0.05); computer applications/ehealth (4.57, 0.05); general practice (4.57, 0.05); university students (4.57, 0.05); mobile application rating scale (4.57, 0.05); community mental health (4.57, 0.05); psychiatric care (4.57, 0.05); personal sensing (4.57, 0.05); phone (4.57, 0.05); technology ethics (4.57, 0.05); software framework (4.57, 0.05); ptsd (4.57, 0.05); clinical services (4.57, 0.05); peer support (4.57, 0.05); rheumatology (4.57, 0.05); sts (science and technology studies) (4.57, 0.05); mental health and illness (4.57, 0.05); ecological momentary assessment (4.57, 0.05); hearing (4.57, 0.05); deaf persons (4.57, 0.05); smartphone sensor (4.57, 0.05); posttraumatic stress disorder (4.57, 0.05); smartphone apps (4.57, 0.05); remote healthcare (4.57, 0.05); refugee (4.57, 0.05); first-episode psychosis (4.57, 0.05); randomized clinical trial (4.57, 0.05); primary care-mental health integration (4.57, 0.05); technology assessment (4.57, 0.05); accidents and injuries (4.57, 0.05); regulation (4.57, 0.05); clinical high risk of psychosis (4.57, 0.05); syria (4.57, 0.05); traumatization (4.57, 0.05); privacy (4.57, 0.05); shared decision making (4.57, 0.05); sign language (4.57, 0.05); digital tools (4.57, 0.05); iphone (4.57, 0.05); proper distance (4.57, 0.05); mobile health technology (4.57, 0.05); software development (4.57, 0.05); digital medicine (4.57, 0.05); posttraumatic stress (4.57, 0.05); anxiety (4.4, 0.05); depression (4.38, 0.05); mobile applications (4.21, 0.05); app (4.03, 0.05); mobile app (4.03, 0.05); digital (4.03, 0.05); telemedicine (3.79, 0.1); mobile phone (3.55, 0.1); e-mental health (3.55, 0.1); mobile mental health (3.06, 0.1); health informatics (3.06, 0.1); social support (2.95, 0.1); medical informatics (2.95, 0.1); ehealth literacy (2.73, 0.1); review (2.73, 0.1); stress (2.37, 0.5); treatment (2.37, 0.5); education (2.3, 0.5); health information (2.3, 0.5); digital divide (2.3, 0.5); chronic disease (2.08, 0.5); older adults (2.08, 0.5); obesity (2.08, 0.5); task shifting (2.01, 0.5); medical device (2.01, 0.5); smartphones (2.01, 0.5); longitudinal studies (2.01, 0.5); well-being (2.01, 0.5); community health worker (2.01, 0.5); drugs (2.01, 0.5) |
| **#2 health literacy** | 56 | 2013 | health literacy; psychometrics; classical test theory; item response theory; adolescents; cancer survivors; digitization; nursing student; breast neoplasms; social support \| ehealth literacy; communication; chronic illness; self efficacy; online food marketing; cluster analysis; medical imaging; consumer health; physical activity; cultural sensitivity | health literacy (39.76, 1.0E-4); ehealth literacy (32.53, 1.0E-4); eheals (22.59, 1.0E-4); literacy (16.18, 1.0E-4); mhealth (15.4, 1.0E-4); internet (9.02, 0.005); ehealth literacy scale (6.92, 0.01); mental health (6.56, 0.05); intervention research (6.13, 0.05); online food marketing (6.13, 0.05); health information seeking (6.13, 0.05); college (6.13, 0.05); electronic (6.13, 0.05); scales (6.13, 0.05); hungary (6.13, 0.05); nurses (6.13, 0.05); sri lanka (6.13, 0.05); electronic health literacy (6.13, 0.05); dietary (6.13, 0.05); nursing education (6.13, 0.05); heart failure (6.13, 0.05); psychmetric evaluation (6.13, 0.05); media literacy (6.13, 0.05); classical test theory (6.13, 0.05); owner (6.13, 0.05); persons living with hiv (6.13, 0.05); item-response theory (6.13, 0.05); cardiac events (6.13, 0.05); item response theory (6.13, 0.05); nursing students (6.13, 0.05); e-nurse (6.13, 0.05); mediation model (6.13, 0.05); aids (6.13, 0.05); learning (6.13, 0.05); self-assessment (6.13, 0.05); reliable web sites for health information (6.13, 0.05); eq-5d-5l (6.13, 0.05); low income (6.13, 0.05); patient education (5.83, 0.05); depression (4.12, 0.05); telemedicine (3.92, 0.05); adolescent (3.84, 0.05); hearing loss (3.45, 0.1); arab (3.45, 0.1); cluster analysis (3.45, 0.1); hls-eu (3.45, 0.1); consumer health (3.45, 0.1); breast neoplasms (3.45, 0.1); digitization (3.45, 0.1); prenatal education (3.45, 0.1); asthma education (3.45, 0.1); medical imaging (3.45, 0.1); perceived competence (3.45, 0.1); psychometrics (3.45, 0.1); carpal tunnel surgery (3.45, 0.1); social cognitive theory (3.45, 0.1); cultural sensitivity (3.45, 0.1); perceived information quality (3.45, 0.1); information-seeking (3.45, 0.1); european health literacy survey (3.45, 0.1); cognitive function (3.45, 0.1); health status (3.45, 0.1); deaf (3.45, 0.1); perceived ewom credibility (3.45, 0.1); price perception (3.45, 0.1); kuwait (3.45, 0.1); patient education as topic (3.45, 0.1); nursing student (3.45, 0.1); readability (3.45, 0.1); willingness to purchase online mental health services (3.45, 0.1); telehealth (3.15, 0.1); digital health (3.05, 0.1); ehealth (2.9, 0.1); systematic review (2.86, 0.1); mobile health (2.66, 0.5); self-management (2.57, 0.5); disease (2.5, 0.5); online health communities (2.5, 0.5); information seeking behavior (2.5, 0.5); e-health literacy (2.5, 0.5); informatics (2.5, 0.5); carers (2.5, 0.5); smartphone (2.09, 0.5); social support (1.93, 0.5); consumer health informatics (1.92, 0.5); pediatrics (1.92, 0.5); reliability (1.92, 0.5); disclosure (1.92, 0.5); skill (1.92, 0.5); self efficacy (1.92, 0.5); nursing (1.92, 0.5); primary care (1.89, 0.5); anxiety (1.7, 0.5); cancer (1.6, 0.5); technology (1.59, 0.5); communication (1.54, 0.5); health-related quality of life (1.51, 0.5); validity (1.51, 0.5); health education (1.51, 0.5); support (1.51, 0.5) |
| **#3 smoking cessation** | 53 | 2013 | mobile health; telemedicine; health care evaluation mechanisms; interviews; topic; data collection; inhalation therapy; smartphone; digital behavior change intervention; mobile phones \| mental health; eating disorders; mobile applications; focus groups; health education; public health practice; oncologists; inhalation therapy; smartphone; digital behavior change intervention | smoking cessation (14.44, 0.001); smoking (9.62, 0.005); electronic health record (6.69, 0.01); affective disorders (4.8, 0.05); e-alliance (4.8, 0.05); personal health record (4.8, 0.05); patient self-management (4.8, 0.05); aboriginal (4.8, 0.05); decision trees (4.8, 0.05); translational research (4.8, 0.05); factorial experiment (4.8, 0.05); transparency (4.8, 0.05); pedagogical agent (4.8, 0.05); codesign (4.8, 0.05); intervention optimization (4.8, 0.05); indigenous (4.8, 0.05); health information management (4.8, 0.05); data collection (4.8, 0.05); working alliance (4.8, 0.05); electronic mail (4.8, 0.05); text-messaging (4.8, 0.05); randomised controlled trial (4.8, 0.05); self-efficacy (4.8, 0.05); indigenous health (4.8, 0.05); therapeutic alliance (4.8, 0.05); patient passport (4.8, 0.05); web intervention (4.8, 0.05); free-text answers (4.8, 0.05); open notes (4.8, 0.05); work environment (4.8, 0.05); co-occurring disorders (4.8, 0.05); web survey (4.8, 0.05); self-guided intervention (4.8, 0.05); humans (4.8, 0.05); health care professionals (4.8, 0.05); cardiac rehabilitation (4.8, 0.05); healthcare service innovation and it (4.8, 0.05); first nations (4.8, 0.05); mobile phones (4.8, 0.05); health care evaluation mechanisms (4.8, 0.05); tobacco (4.8, 0.05); patient accessible electronic health records (4.8, 0.05); patient dropouts (4.8, 0.05); embodied conversational agent (4.8, 0.05); realist review (4.8, 0.05); logistic regression (4.8, 0.05); chatbot (4.8, 0.05); pregnant smokers (4.8, 0.05); interviews as topic (4.8, 0.05); mobile health (3.95, 0.05); breast cancer (3.46, 0.1); cancer (3.18, 0.1); communication (2.99, 0.1); risk (2.74, 0.1); medical informatics (2.6, 0.5); cognitive behavioral therapy (2.6, 0.5); ehealth literacy (2.41, 0.5); patient-centred care (2.22, 0.5); forensic psychiatry (2.22, 0.5); survivors (2.22, 0.5); activation (2.22, 0.5); energy balance (2.22, 0.5); general health questionnaire (2.22, 0.5); implementation outcomes (2.22, 0.5); service (2.22, 0.5); tobacco cessation (2.22, 0.5); older people (2.22, 0.5); e-learning (2.22, 0.5); cardiovascular disease (2.22, 0.5); integrated care (2.22, 0.5); chronic patients (2.22, 0.5); design (2.22, 0.5); focus groups (2.22, 0.5); registered nurses (2.22, 0.5); health information exchange (2.22, 0.5); oral & maxillofacial (2.22, 0.5); digital behavior change intervention (2.22, 0.5); time pressure (2.22, 0.5); search (2.22, 0.5); game design theory (2.22, 0.5); case study (2.22, 0.5); vision (2.22, 0.5); data mining (2.22, 0.5); telecommunications (2.22, 0.5); motivational intervention (2.22, 0.5); model (2.22, 0.5); implementation strategies (2.22, 0.5); life style (2.22, 0.5); usability and feasibility testing (2.22, 0.5); virtual reality (2.22, 0.5); program evaluation (2.22, 0.5); design process (2.22, 0.5); ehealth services (2.22, 0.5); interoperability (2.22, 0.5); eating disorders (2.22, 0.5); device (2.22, 0.5); information and communication technology (2.22, 0.5); technology development (2.22, 0.5); telemonitoring (2.22, 0.5); survivorship (2.22, 0.5) |
| **#4 physical activity** | 47 | 2012 | physical activity; controlled trial; chronic disease; healthy eating; lifestyle intervention; health behaviours; self-regulation; measurement; sedentary behaviour; public health \| self-regulation; sedentary behaviour; implementation intention; online program; mobile apps; digital behavior change intervention; attitudes; mental health; controlled intervention trial; african american women | physical activity (28.44, 1.0E-4); exercise (16.64, 1.0E-4); self-regulation (15.41, 1.0E-4); fitness (11.38, 0.001); sedentary behaviour (11.38, 0.001); sedentary behavior (7.69, 0.01); promotion (7.69, 0.01); wearable (6.08, 0.05); measurement (5.69, 0.05); guideline (5.69, 0.05); functional assessment (5.69, 0.05); quantified self (5.69, 0.05); behavioral medicine (5.69, 0.05); dietary assessment (5.69, 0.05); personal health monitoring (5.69, 0.05); physical fitness (5.69, 0.05); sodium policy (5.69, 0.05); markov chain (5.69, 0.05); treatment engagement (5.69, 0.05); college students (5.69, 0.05); statement (5.69, 0.05); lifestyle (5.69, 0.05); antimicrobial resistance (5.69, 0.05); style (5.69, 0.05); facility (5.69, 0.05); log data (5.69, 0.05); implementation intention (5.69, 0.05); physical activity participation (5.69, 0.05); public health (5.69, 0.05); low back pain (5.69, 0.05); sodium (5.69, 0.05); social network site (5.69, 0.05); physiotherapy (5.69, 0.05); behaviour change (5.69, 0.05); perceived barrier (5.69, 0.05); african american women (5.69, 0.05); attitudes and behaviours (5.69, 0.05); data analytics (5.69, 0.05); maternal health (5.69, 0.05); aged (5.69, 0.05); healthy diet (5.69, 0.05); depression (5.17, 0.05); telehealth (3.95, 0.05); technology (3.46, 0.1); mental health (3.36, 0.1); mobile health (3.34, 0.1); self-management (3.22, 0.1); quality of life (3.1, 0.1); health literacy (3.1, 0.1); happiness (3.03, 0.1); study protocol (3.03, 0.1); controlled intervention trial (3.03, 0.1); rehabilitation (3.03, 0.1); behavior change technique (3.03, 0.1); value orientation (3.03, 0.1); type 2 diabetes (3.03, 0.1); neoplasms (3.03, 0.1); healthy aging (3.03, 0.1); internet of things (3.03, 0.1); latinas (3.03, 0.1); dietary behavior (3.03, 0.1); connected health (3.03, 0.1); tailored or targeted interventions (3.03, 0.1); digital behavior change intervention (3.03, 0.1); impact (3.03, 0.1); survivor (3.03, 0.1); behaviour-change techniques (3.03, 0.1); healthy lifestyle (3.03, 0.1); social-cognitive predictor (3.03, 0.1); life style intervention (3.03, 0.1); ehealth intervention (3.03, 0.1); mediation (3.03, 0.1); cost (3.03, 0.1); health behavior (2.68, 0.5); smartphone (2.62, 0.5); chronic disease (2.33, 0.5); anxiety (2.13, 0.5); youth (2.11, 0.5); or hip (2.11, 0.5); child health (2.11, 0.5); medical technology (2.11, 0.5); health behaviours (2.11, 0.5); weight management (2.11, 0.5); blood glucose (2.11, 0.5); knee and (2.11, 0.5); pragmatic randomized controlled trial (2.11, 0.5); lifestyle intervention (2.11, 0.5); online program (2.11, 0.5); hypertension prevention (2.11, 0.5); osteoarthritis (2.11, 0.5); behavioral theories (2.11, 0.5); persuasive communication (2.11, 0.5); older (2.11, 0.5); weight loss maintenance (2.11, 0.5); behavioural change techniques (2.11, 0.5); adult (2.11, 0.5); print-delivered intervention (2.11, 0.5); metaanalysis (2.11, 0.5); blood pressure (2.11, 0.5); text message (2.11, 0.5) |
| **#5 obesity** | 46 | 2014 | telemedicine; digital health; hypertension prevention; medical technology; e-mental health; consort ehealth; reporting standards; relationship; mobile application; communication \| chronic disease; physical activity; controlled trial; health behaviours; healthy eating; lifestyle intervention; postpartum women; mental health; lifestyle management; user engagement | obesity (12.04, 0.001); internet (8.78, 0.005); overweight (8.62, 0.005); risk (7.2, 0.01); weight loss (7.2, 0.01); weight management (6.35, 0.05); weight (6.35, 0.05); review (5.51, 0.05); focused attention (4.99, 0.05); mediators (4.99, 0.05); patient-generated health data (4.99, 0.05); childhood obesity (4.99, 0.05); susceptibility (4.99, 0.05); mobile technology (4.99, 0.05); behavior interventions (4.99, 0.05); nutrition (4.99, 0.05); moderators (4.99, 0.05); attentive eating (4.99, 0.05); scientific statement (4.99, 0.05); inflammatory bowel disease (4.99, 0.05); bmi (4.99, 0.05); neophobia (4.99, 0.05); personal health information (4.99, 0.05); responsive parenting intervention (4.99, 0.05); association (4.99, 0.05); 1st (4.99, 0.05); crohn's disease (4.99, 0.05); awareness (4.99, 0.05); questionnaire (4.99, 0.05); ulcerative colitis (4.99, 0.05); heart disease (4.99, 0.05); food intake (4.99, 0.05); primary prevention (4.8, 0.05); health literacy (4.43, 0.05); chronic disease (3.99, 0.05); body weight (3.78, 0.1); mental health (3.34, 0.1); system (3.05, 0.1); self management (3.05, 0.1); knowledge (3.05, 0.1); outcm (3.05, 0.1); cancer (2.88, 0.1); qualitative research (2.53, 0.5); cardiovascular diseases (2.48, 0.5); forensic psychiatry (2.4, 0.5); activation (2.4, 0.5); child (2.4, 0.5); wearable technology (2.4, 0.5); service (2.4, 0.5); tobacco cessation (2.4, 0.5); older people (2.4, 0.5); women (2.4, 0.5); cardiovascular disease (2.4, 0.5); digital health interventions (2.4, 0.5); self report (2.4, 0.5); measurement development (2.4, 0.5); body mass index (2.4, 0.5); neoplasms (2.4, 0.5); human technology interaction (2.4, 0.5); motivational interviewing (2.4, 0.5); tailored or targeted interventions (2.4, 0.5); smartphone application (2.4, 0.5); search (2.4, 0.5); game design theory (2.4, 0.5); embodiment (2.4, 0.5); self-help devices (2.4, 0.5); dimensional measurement accuracy (2.4, 0.5); case study (2.4, 0.5); motivational intervention (2.4, 0.5); model (2.4, 0.5); life style (2.4, 0.5); usability and feasibility testing (2.4, 0.5); virtual reality (2.4, 0.5); technology development (2.4, 0.5); lifestyle management (2.4, 0.5); health outcm (2.4, 0.5); body height (2.4, 0.5); wearable electronic devices (2.4, 0.5); postpartum women (2.4, 0.5); europe (2.4, 0.5); personal health (2.4, 0.5); cognitive behavioral therapy (2.36, 0.5); ehealth literacy (2.18, 0.5); health promotion (2.03, 0.5); usage (2.03, 0.5); behavior change (2.03, 0.5); adolescent (2.03, 0.5); mobile apps (2.01, 0.5); telemedicine (1.87, 0.5); health information (1.84, 0.5); adolescents (1.84, 0.5); digital divide (1.84, 0.5); literacy (1.84, 0.5); adherence (1.78, 0.5); psychosis (1.67, 0.5); older adults (1.67, 0.5); prevention (1.66, 0.5); or hip (1.52, 0.5); research and development (1.52, 0.5); medical technology (1.52, 0.5) |
| **#6 telehealth** | 42 | 2014 | telemedicine; consumer satisfaction; ehealth services; health information exchange; interoperability; smartphone; healthcare system; telehealth technology; underserved area; mental health \| digital divide; patient portals; health; social determinants; health literacy; health equity; smartphone; healthcare system; telehealth technology; underserved area | telehealth (17.12, 1.0E-4); telemedicine (15.79, 1.0E-4); digital divide (6.85, 0.01); substance use (6.38, 0.05); online health (5.01, 0.05); extended release naltrexone (5.01, 0.05); telehomecare (5.01, 0.05); consumer satisfaction (5.01, 0.05); health care delivery (5.01, 0.05); reduced drinking (5.01, 0.05); ehealth digital intervention (5.01, 0.05); typology (5.01, 0.05); european union (5.01, 0.05); health disparities (5.01, 0.05); angels (5.01, 0.05); disability (5.01, 0.05); contingency management (5.01, 0.05); underserved area (5.01, 0.05); e-health policy (5.01, 0.05); treatment sbirt (5.01, 0.05); sms (5.01, 0.05); electronic medical records (5.01, 0.05); client heterogeneity (5.01, 0.05); behavioral counseling intervention (5.01, 0.05); quality (5.01, 0.05); identification test audit (5.01, 0.05); online psychotherapy (5.01, 0.05); techno-scientific imaginaries (5.01, 0.05); teleconference (5.01, 0.05); national epidemiologic survey (5.01, 0.05); knowledge assessment (5.01, 0.05); gestational diabetes (5.01, 0.05); digital intervention (5.01, 0.05); behavioral activation (5.01, 0.05); technology-enabled pharmacy (5.01, 0.05); wearable sensors (5.01, 0.05); mental health (3.28, 0.1); policy (3.07, 0.1); patient portals (3.07, 0.1); anxiety (3.02, 0.1); adherence (2.68, 0.5); implementation (2.68, 0.5); qualitative research (2.51, 0.5); telehealth technology (2.41, 0.5); behavior adherence (2.41, 0.5); trend study (2.41, 0.5); child (2.41, 0.5); china (2.41, 0.5); depressive symptoms (2.41, 0.5); osteoporosis (2.41, 0.5); continuum of care (2.41, 0.5); behavior change technique (2.41, 0.5); socioeconomic factors (2.41, 0.5); self report (2.41, 0.5); dental caries (2.41, 0.5); interactive design (2.41, 0.5); body mass index (2.41, 0.5); mobile app intervention (2.41, 0.5); underserved populations (2.41, 0.5); health technologies (2.41, 0.5); methods (2.41, 0.5); health information exchange (2.41, 0.5); dietary behavior (2.41, 0.5); motivational interviewing (2.41, 0.5); impact (2.41, 0.5); fls (2.41, 0.5); social networking (2.41, 0.5); dimensional measurement accuracy (2.41, 0.5); e-prescribing (2.41, 0.5); secure messaging (2.41, 0.5); mental health care (2.41, 0.5); healthcare system (2.41, 0.5); ehealth services (2.41, 0.5); interoperability (2.41, 0.5); internet health information seeking (2.41, 0.5); hepatitis c virus (2.41, 0.5); virtual care service (2.41, 0.5); health disparity (2.41, 0.5); user interface (2.41, 0.5); life style intervention (2.41, 0.5); body height (2.41, 0.5); social determinants of health (2.41, 0.5); ehealth intervention (2.41, 0.5); telecare (2.41, 0.5); rural population (2.41, 0.5); virtual care (2.41, 0.5); research performance (2.41, 0.5); cost (2.41, 0.5); mixed-methods (2.41, 0.5); cognitive behavioral therapy (2.34, 0.5); review (2.17, 0.5); mobile apps (2, 0.5); hiv (2, 0.5); adolescents (1.83, 0.5); validation (1.83, 0.5); literacy (1.83, 0.5); communication (1.81, 0.5); chronic disease (1.65, 0.5); psychosis (1.65, 0.5); epro systems (1.53, 0.5) |
| **#7 older adults** | 40 | 2009 | mental health; evidence-based treatment; mobile applications; health technology; technophilia; mobile apps; information technology; telehealth technology; frontline care; health equity \| communication; chronic illness; self efficacy; ehealth literacy; electronic health record; health equity; justice theory; behavioral intention; digital divide; online consultation | older adults (8.15, 0.005); depression (6.79, 0.01); information technology (5.16, 0.05); starting-stopping internet use (5.16, 0.05); fairness theory (5.16, 0.05); health-related activities (5.16, 0.05); longitudinal study (5.16, 0.05); s-o-r framework (5.16, 0.05); online consultation (5.16, 0.05); lifestyle improvements (5.16, 0.05); justice theory (5.16, 0.05); technophilia (5.16, 0.05); brand extension theory (5.16, 0.05); health information on the web (5.16, 0.05); mental models (5.16, 0.05); visual design (5.16, 0.05); aging (5.16, 0.05); pelvic floor muscle training (5.16, 0.05); expectation-confirmation model (5.16, 0.05); information and knowledge management (5.16, 0.05); technology readiness (5.16, 0.05); o2o commerce (5.16, 0.05); interviews (5.16, 0.05); behavioral intention (5.16, 0.05); e-health/m-health (5.16, 0.05); online healthcare services (5.16, 0.05); mixed methods design (5.16, 0.05); urinary incontinence (5.16, 0.05); health communication (5.09, 0.05); information systems (5.09, 0.05); technology (5.06, 0.05); communication (4.56, 0.05); self-management (4.23, 0.05); gamification (4.07, 0.05); digital divide (3.86, 0.05); policy (3.31, 0.1); electronic health records (3.31, 0.1); software design (3.31, 0.1); anxiety (2.8, 0.1); telehealth technology (2.54, 0.5); well-being (2.54, 0.5); trend study (2.54, 0.5); young (2.54, 0.5); wearable technology (2.54, 0.5); happiness (2.54, 0.5); study protocol (2.54, 0.5); general health questionnaire (2.54, 0.5); mental healthcare (2.54, 0.5); user-centred design (2.54, 0.5); women (2.54, 0.5); socioeconomic factors (2.54, 0.5); value orientation (2.54, 0.5); measurement development (2.54, 0.5); educational technology (2.54, 0.5); frailty (2.54, 0.5); human technology interaction (2.54, 0.5); registered nurses (2.54, 0.5); cognitive function (2.54, 0.5); seeking (2.54, 0.5); user acceptance (2.54, 0.5); health technology (2.54, 0.5); time pressure (2.54, 0.5); health app (2.54, 0.5); embodiment (2.54, 0.5); unified theory (2.54, 0.5); self-help devices (2.54, 0.5); self-care (2.54, 0.5); persuasive game design (2.54, 0.5); video games (2.54, 0.5); divide (2.54, 0.5); secure messaging (2.54, 0.5); nurse-patient relationships (2.54, 0.5); prenatal care (2.54, 0.5); healthy lifestyle (2.54, 0.5); internet health information seeking (2.54, 0.5); virtual care service (2.54, 0.5); health disparity (2.54, 0.5); age difference (2.54, 0.5); antenatal care (2.54, 0.5); bass model (2.54, 0.5); wearable electronic devices (2.54, 0.5); diffusion of innovation (2.54, 0.5); social determinants of health (2.54, 0.5); nurse-patient interaction (2.54, 0.5); ghq (2.54, 0.5); virtual care (2.54, 0.5); telenursing (2.54, 0.5); adherence (2.48, 0.5); qualitative research (2.36, 0.5); older adult (2.27, 0.5); social support (2.17, 0.5); telemedicine (2.09, 0.5); review (2.01, 0.5); health behavior (1.89, 0.5); adolescents (1.69, 0.5); validation (1.69, 0.5); literacy (1.69, 0.5); psychological stress (1.65, 0.5); workload (1.65, 0.5); online survey (1.65, 0.5) |
| **#8 cancer** | 28 | 2015 | life; health-related quality; cancer survivors; patient-reported outcome measures; implementation; cognitive behavioral therapy; erectile function; mental health; measurement properties; prostate cancer survivors \| physical activity; internet; rehabilitation; things; connected health; cognitive behavioral therapy; erectile function; mental health; measurement properties; prostate cancer survivors | cancer (27.43, 1.0E-4); oncology (19.24, 1.0E-4); adoption (12.46, 0.001); quality of life (8.57, 0.005); breast cancer survivors (7.1, 0.01); online (7.1, 0.01); palliative care (6.22, 0.05); spontaneous internet use (6.22, 0.05); patient-reported outcome measures (6.22, 0.05); fear of cancer recurrence (6.22, 0.05); measurement properties (6.22, 0.05); colorectal cancer (6.22, 0.05); meditation (6.22, 0.05); web-based (6.22, 0.05); international index of erectile function (6.22, 0.05); patient-reported outcome measure (prom) (6.22, 0.05); psycho-oncology (6.22, 0.05); cancer patients (6.22, 0.05); supportive care (6.22, 0.05); cosmin (6.22, 0.05); psychological impact (6.22, 0.05); chemotherapy (6.22, 0.05); eortc qlq-cr29 (6.22, 0.05); incurable cancer (6.22, 0.05); guided internet use (6.22, 0.05); measurement property (6.22, 0.05); e-health impact questionnaire (6.22, 0.05); health-related quality of life (6, 0.05); breast cancer (6, 0.05); mindfulness (6, 0.05); cancer survivors (4.54, 0.05); attrition (4.54, 0.05); self-help intervention (3.54, 0.1); survivors (3.54, 0.1); energy balance (3.54, 0.1); online social networking (3.54, 0.1); rehabilitation (3.54, 0.1); fear of recurrence (3.54, 0.1); self-help (3.54, 0.1); cancer survivorship (3.54, 0.1); psychometrics (3.54, 0.1); interactive design (3.54, 0.1); acceptance and commitment therapy (act) (3.54, 0.1); self-help groups (3.54, 0.1); internet of things (3.54, 0.1); mobile app intervention (3.54, 0.1); connected health (3.54, 0.1); survivor (3.54, 0.1); social networking (3.54, 0.1); eu (3.54, 0.1); internet psychosocial intervention (3.54, 0.1); portugal (3.54, 0.1); patient activation (3.54, 0.1); colorectal cancer survivors (3.54, 0.1); spinal fusion (3.54, 0.1); randomized controlled trial protocol (3.54, 0.1); prostate cancer survivors (3.54, 0.1); user interface (3.54, 0.1); survivorship (3.54, 0.1); telehealth (3, 0.1); technology (2.63, 0.5); rct (2.59, 0.5); trust (2.59, 0.5); monitoring (2.59, 0.5); clinical trial (2.59, 0.5); pst (2.59, 0.5); dialysis (2.59, 0.5); print-delivered intervention (2.59, 0.5); reporting standards (2.59, 0.5); cbt (2.59, 0.5); mobile health (2.54, 0.5); health literacy (2.36, 0.5); social support (2.06, 0.5); medical informatics (2.06, 0.5); physical activity (2.03, 0.5); self-management (2.03, 0.5); hair cortisol (2, 0.5); web-based intervention (2, 0.5); wearable (2, 0.5); reliability (2, 0.5); consort ehealth (2, 0.5); e-health (2, 0.5); compliance (2, 0.5); smartphone (1.99, 0.5); primary care (1.8, 0.5); mhealth (1.66, 0.5); anxiety (1.62, 0.5); validity (1.58, 0.5); caregivers (1.58, 0.5); user experience (1.58, 0.5); communication (1.44, 0.5); qualitative research (1.34, 0.5); cost-effectiveness (1.27, 0.5); treatment (1.27, 0.5); outcm (1.27, 0.5); ehealth literacy (1.16, 0.5); mobile apps (1.07, 0.5); hiv (1.07, 0.5); intervention (1.03, 0.5); education (0.98, 0.5) |
| **#9 health information** | 28 | 2013 | health information; healthcare professional; personal experiences; age difference; adult; unified theory; user acceptance; cultural sensitivity; health education; ehealth access \| medical informatics; telemedicine; systematic review; consumer health information; cultural sensitivity; health education; ehealth access; doctor-seeking behavior; health information; type | health information (12.16, 0.001); cross-sectional study (9.25, 0.005); trust (9.25, 0.005); decision-making (9.25, 0.005); internet (8.28, 0.005); norway (7.6, 0.01); mhealth (7.07, 0.01); patient education (6.49, 0.05); systematic map (6.48, 0.05); patient experiences (6.48, 0.05); income (6.48, 0.05); health care utilization (6.48, 0.05); information seeking (6.48, 0.05); distributed (6.48, 0.05); inequalities (6.48, 0.05); personal experiences (6.48, 0.05); ehealth access (6.48, 0.05); ehealth systems (6.48, 0.05); toothache (6.48, 0.05); pediatric surgery (6.48, 0.05); multivariate logistic regression (6.48, 0.05); predictive techniques (6.48, 0.05); diabetes mellitus type 2 (6.48, 0.05); diabetes mellitus type 1 (6.48, 0.05); blockchain (6.48, 0.05); systematic literature review (6.48, 0.05); regional australia (6.48, 0.05); healthcare professional (6.48, 0.05); western downs region (6.48, 0.05); general surgery (6.48, 0.05); medical informatics (5.82, 0.05); mental health (5.47, 0.05); communication (5.08, 0.05); diabetes (5, 0.05); young (3.79, 0.1); online social networking (3.79, 0.1); consumer health (3.79, 0.1); china (3.79, 0.1); doctor-seeking behavior (3.79, 0.1); drugs (3.79, 0.1); carpal tunnel surgery (3.79, 0.1); social cognitive theory (3.79, 0.1); pharmaceuticals (3.79, 0.1); self-help groups (3.79, 0.1); cultural sensitivity (3.79, 0.1); program (3.79, 0.1); underserved populations (3.79, 0.1); stigma (3.79, 0.1); therapy (3.79, 0.1); seeking (3.79, 0.1); user acceptance (3.79, 0.1); unified theory (3.79, 0.1); data mining (3.79, 0.1); divide (3.79, 0.1); spinal fusion (3.79, 0.1); age difference (3.79, 0.1); internet information (3.79, 0.1); information science (3.79, 0.1); emotion (3.79, 0.1); rural population (3.79, 0.1); readability (3.79, 0.1); depression (3.44, 0.1); education (3.27, 0.1); literacy (3.27, 0.1); online health communities (2.83, 0.1); e-health literacy (2.83, 0.1); telehealth (2.63, 0.5); digital health (2.55, 0.5); behavior (2.23, 0.5); disclosure (2.23, 0.5); skill (2.23, 0.5); self efficacy (2.23, 0.5); cross-sectional survey (2.23, 0.5); mobile health (2.22, 0.5); cell phone (1.8, 0.5); health education (1.8, 0.5); support (1.8, 0.5); primary care (1.58, 0.5); knowledge (1.48, 0.5); electronic health record (1.48, 0.5); anxiety (1.42, 0.5); adherence (1.26, 0.5); implementation (1.26, 0.5); survey (1.22, 0.5); qualitative research (1.18, 0.5); cognitive behavioral therapy (1.1, 0.5); review (1.02, 0.5); older adult (1.01, 0.5); mobile apps (0.94, 0.5); hiv (0.94, 0.5); digital divide (0.86, 0.5); validation (0.86, 0.5); consumer health information (0.84, 0.5); engagement (0.84, 0.5); exercise (0.84, 0.5); prevention (0.84, 0.5); health literacy (0.78, 0.5); chronic disease (0.78, 0.5); psychosis (0.78, 0.5); older adults (0.78, 0.5) |
| **#10 psychosis** | 23 | 2012 | digital health; mobile health; consumer protection; advertising standards; digital mental health interventions; digital health applications; think-aloud test; mental health; controlled intervention trial; nurse-patient relationships \| schizophrenia; think-aloud test; mental health; controlled intervention trial; nurse-patient relationships; health care policy; online health care service; behavior adherence; antenatal care; artificial intelligence | psychosis (14.41, 0.001); schizophrenia (11.28, 0.001); learning outcomes (6.84, 0.01); mild cognitive impairment (6.84, 0.01); carer (6.84, 0.01); public and patient involvement (6.84, 0.01); patient perspectives (6.84, 0.01); home care nursing (6.84, 0.01); tablet (6.84, 0.01); patient-centeredness (6.84, 0.01); autonomic (6.84, 0.01); coproduction (6.84, 0.01); family caregivers (6.84, 0.01); diabetes (5.66, 0.05); behavior adherence (4.14, 0.05); health care professional-patient interaction (4.14, 0.05); family (4.14, 0.05); controlled intervention trial (4.14, 0.05); digital health interventions (4.14, 0.05); heuristic evaluation (4.14, 0.05); mobile interventions (4.14, 0.05); serious mental illness (4.14, 0.05); digital health applications (4.14, 0.05); online health care service (4.14, 0.05); healthy aging (4.14, 0.05); online communication (4.14, 0.05); usability evaluation (4.14, 0.05); digital mental health interventions (4.14, 0.05); consumer protection (4.14, 0.05); advertising standards (4.14, 0.05); health app (4.14, 0.05); think-aloud test (4.14, 0.05); nurse-patient relationships (4.14, 0.05); prenatal care (4.14, 0.05); illness management (4.14, 0.05); social-cognitive predictor (4.14, 0.05); antenatal care (4.14, 0.05); lifestyle management (4.14, 0.05); system usability scale (4.14, 0.05); bass model (4.14, 0.05); participatory research (4.14, 0.05); mobile health (mhealth) (4.14, 0.05); diffusion of innovation (4.14, 0.05); postpartum women (4.14, 0.05); mediation (4.14, 0.05); nurse-patient interaction (4.14, 0.05); telenursing (4.14, 0.05); research performance (4.14, 0.05); internet (3.41, 0.1); legislation (3.16, 0.1); health care policy (3.16, 0.1); weight (3.16, 0.1); conversational agents (3.16, 0.1); international collaboration (3.16, 0.1); chatbots (3.16, 0.1); carers (3.16, 0.1); depression (2.87, 0.1); surveillance (2.54, 0.5); wearable (2.54, 0.5); app (2.54, 0.5); information and communication technologies (2.54, 0.5); telehealth (2.19, 0.5); dementia (2.1, 0.5); framework (2.1, 0.5); qualitative (2.1, 0.5); usability (2.1, 0.5); paradigm (2.1, 0.5); nonpharmacological interventions (2.1, 0.5); caregivers (2.1, 0.5); behavioral intervention technology (2.1, 0.5); physical activity (1.79, 0.5); electronic health records (1.77, 0.5); artificial intelligence (1.77, 0.5); health policy (1.77, 0.5); machine learning (1.5, 0.5); smartphone (1.45, 0.5); mobile applications (1.28, 0.5); anxiety (1.18, 0.5); cancer (1.12, 0.5); adherence (1.05, 0.5); communication (1.05, 0.5); social support (0.91, 0.5); cognitive behavioral therapy (0.91, 0.5); mental health (0.9, 0.5); ehealth literacy (0.85, 0.5); education (0.8, 0.5); validation (0.8, 0.5); mobile apps (0.78, 0.5); hiv (0.78, 0.5); digital health (0.72, 0.5); health information (0.71, 0.5); adolescents (0.71, 0.5); chronic illness (0.71, 0.5); digital divide (0.71, 0.5); literacy (0.71, 0.5); chronic disease (0.65, 0.5); older adults (0.65, 0.5); obesity (0.65, 0.5); review (0.59, 0.5); mobile phone (0.58, 0.5) |
| **#11 usability testing** | 16 | 2010 | mental health; iterative prototype testing; ambulatory biofeedback; mobile health; design; design science; borderline personality disorder; emotional awareness; user; think-aloud test \| outpatient care; care; health information technology; web-application; inpatient; continuity; medication reconciliation; iterative user-centered design; inhalation therapy; think-aloud test | usability testing (10.22, 0.005); work (7.43, 0.01); emotional awareness (6.97, 0.01); borderline personality disorder (6.97, 0.01); user centered design (6.97, 0.01); usability benchmarking (6.97, 0.01); quality improvement (6.97, 0.01); iterative prototype testing (6.97, 0.01); think aloud (6.97, 0.01); hispanic (6.97, 0.01); design science (6.97, 0.01); web-application (6.97, 0.01); feasibility (6.97, 0.01); ambulatory biofeedback (6.97, 0.01); breast cancer survivor (6.97, 0.01); risk modelling (6.97, 0.01); usability task metrics (6.97, 0.01); inpatient and outpatient care (6.97, 0.01); asthma action plans (6.97, 0.01); continuity of care (6.97, 0.01); sus (6.97, 0.01); medication reconciliation (6.97, 0.01); surveys and questionnaires (6.97, 0.01); multi-disciplinary approach (6.97, 0.01); computerized decision support systems (6.97, 0.01); iterative user-centered design (6.97, 0.01); human centred design (6.97, 0.01); asthma (6.58, 0.05); family (4.27, 0.05); occupation (4.27, 0.05); heuristic evaluation (4.27, 0.05); type 2 diabetes (4.27, 0.05); methods (4.27, 0.05); usability evaluation (4.27, 0.05); occupational exposure (4.27, 0.05); think-aloud test (4.27, 0.05); system usability scale (4.27, 0.05); mixed-methods (4.27, 0.05); epro systems (3.28, 0.1); pharmacy (3.28, 0.1); inhalation therapy (3.28, 0.1); online survey (3.28, 0.1); electronic systems (3.28, 0.1); electronic patient-reported outcomes (3.28, 0.1); health apps (3.28, 0.1); pros (3.28, 0.1); eprom (3.28, 0.1); epros (3.28, 0.1); pharmacy practice research (3.28, 0.1); carers (3.28, 0.1); depression (2.68, 0.5); science (2.66, 0.5); surveillance (2.66, 0.5); health information technology (2.66, 0.5); pregnancy (2.66, 0.5); care (2.66, 0.5); patient engagement (2.66, 0.5); telemedicine (2.55, 0.5); information (2.22, 0.5); framework (2.22, 0.5); health-related quality of life (2.22, 0.5); universities (2.22, 0.5); usability (2.22, 0.5); paradigm (2.22, 0.5); nonpharmacological interventions (2.22, 0.5); caregivers (2.22, 0.5); behavioral intervention technology (2.22, 0.5); system (1.88, 0.5); students (1.88, 0.5); self management (1.88, 0.5); outcm (1.88, 0.5); systematic review (1.86, 0.5); physical activity (1.67, 0.5); self-management (1.67, 0.5); health literacy (1.61, 0.5); copd (1.61, 0.5); health promotion (1.38, 0.5); evaluation (1.38, 0.5); older adult (1.38, 0.5); smartphone (1.36, 0.5); primary care (1.23, 0.5); mobile phone (1.19, 0.5); anxiety (1.1, 0.5); cancer (1.04, 0.5); psychosis (1.03, 0.5); adherence (0.98, 0.5); communication (0.98, 0.5); implementation (0.98, 0.5); digital divide (0.89, 0.5); validation (0.89, 0.5); social support (0.85, 0.5); medical informatics (0.85, 0.5); cognitive behavioral therapy (0.85, 0.5); mhealth (0.85, 0.5); ehealth literacy (0.79, 0.5); review (0.79, 0.5); telehealth (0.79, 0.5); mobile apps (0.73, 0.5); hiv (0.73, 0.5); education (0.67, 0.5) |
| **#12 msm** | 13 | 2013 | noncommunicable diseases; health policy; qualitative research; disease management; sub-saharan africa; implementation science; digital divide; health information; medication adherence; scalability \| implementation; young men; sustainability; intervention development; scalability; systematic review; intervention; digital divide; health information; medication adherence | msm (17.32, 1.0E-4); hiv (10.81, 0.005); sexual risk-taking (7.96, 0.005); sub-saharan africa (7.96, 0.005); keep it up! (7.96, 0.005); suicide (7.96, 0.005); social norms (7.96, 0.005); men who have sex with men (msm) (7.96, 0.005); hiv prevention (7.96, 0.005); sti (7.96, 0.005); highly active antiretroviral therapy (7.96, 0.005); transgender (7.96, 0.005); character identification (7.96, 0.005); hiv/aids intervention (7.96, 0.005); ehealth engagement measurement (7.96, 0.005); intervention acceptability (7.96, 0.005); hiv/aids (5.22, 0.05); medication adherence (5.22, 0.05); implementation science (4.22, 0.05); online health information seeking (4.22, 0.05); science (3.57, 0.1); disease management (3.57, 0.1); scalability (3.57, 0.1); care (3.57, 0.1); homelessness (3.57, 0.1); young men who have sex with men (3.57, 0.1); sustainability (3.57, 0.1); patient engagement (3.57, 0.1); information (3.11, 0.1); intervention development (3.11, 0.1); work (3.11, 0.1); system (2.74, 0.1); self management (2.74, 0.1); outcm (2.74, 0.1); patient portals (2.74, 0.1); health policy (2.74, 0.1); mental health (2.6, 0.5); intervention (2.44, 0.5); noncommunicable diseases (2.19, 0.5); older adult (2.19, 0.5); prevention (1.98, 0.5); health information (1.63, 0.5); adolescents (1.63, 0.5); digital divide (1.63, 0.5); telemedicine (1.56, 0.5); telehealth (1.25, 0.5); digital health (1.21, 0.5); qualitative research (1.14, 0.5); mobile health (1.06, 0.5); implementation (1.04, 0.5); physical activity (1.02, 0.5); self-management (1.02, 0.5); health literacy (0.98, 0.5); smartphone (0.83, 0.5); primary care (0.75, 0.5); anxiety (0.67, 0.5); cancer (0.64, 0.5); adherence (0.6, 0.5); communication (0.6, 0.5); social support (0.52, 0.5); medical informatics (0.52, 0.5); cognitive behavioral therapy (0.52, 0.5); ehealth literacy (0.48, 0.5); review (0.48, 0.5); mobile apps (0.44, 1.0); education (0.41, 1.0); chronic illness (0.41, 1.0); validation (0.41, 1.0); literacy (0.41, 1.0); chronic disease (0.37, 1.0); psychosis (0.37, 1.0); older adults (0.37, 1.0); obesity (0.37, 1.0); mhealth (0.36, 1.0); mobile phone (0.33, 1.0); consumer health information (0.33, 1.0); oncology (0.33, 1.0); engagement (0.33, 1.0); exercise (0.33, 1.0); health behavior (0.33, 1.0); e-mental health (0.33, 1.0); technology (0.31, 1.0); health promotion (0.29, 1.0); usage (0.29, 1.0); quality of life (0.29, 1.0); social media (0.29, 1.0); evaluation (0.29, 1.0); mobile applications (0.29, 1.0); behavior change (0.29, 1.0); adolescent (0.29, 1.0); systematic review (0.28, 1.0); machine learning (0.25, 1.0); cancer survivors (0.25, 1.0); motivation (0.25, 1.0); attrition (0.25, 1.0); diabetes (0.25, 1.0); schizophrenia (0.25, 1.0); text messaging (0.25, 1.0); eheals (0.25, 1.0); health (0.25, 1.0) |
| **#13 artificial intelligence** | 11 | 2015 | digital health; artificial intelligence; mobile health; government regulation; medical informatics; conversational agents; global cause; prenatal education; deep learning; doctor-patient relationship \| social support; information; program evaluation; integrated care; chronic patients; telemonitoring; communication technology; global cause; prenatal education; artificial intelligence | artificial intelligence (16.17, 1.0E-4); digital health (9.4, 0.005); congenital heart defects (8.09, 0.005); vision impairment (8.09, 0.005); uncorrected refractive error (8.09, 0.005); population (8.09, 0.005); global cause (8.09, 0.005); blindness (8.09, 0.005); deep learning (8.09, 0.005); machine learning (8.02, 0.005); ethics (5.36, 0.05); doctor-patient relationship (5.36, 0.05); prevalence (5.36, 0.05); integrated care (5.36, 0.05); chronic patients (5.36, 0.05); prenatal education (5.36, 0.05); continuum of care (5.36, 0.05); smartphone application (5.36, 0.05); vision (5.36, 0.05); government regulation (5.36, 0.05); patient education as topic (5.36, 0.05); program evaluation (5.36, 0.05); hepatitis c virus (5.36, 0.05); mortality (5.36, 0.05); information and communication technology (5.36, 0.05); telemonitoring (5.36, 0.05); social support (5.15, 0.05); information seeking behavior (4.34, 0.05); substance use (4.34, 0.05); conversational agents (4.34, 0.05); chatbots (4.34, 0.05); challenges (4.34, 0.05); primary care (3.84, 0.1); consumer health informatics (3.7, 0.1); elderly (3.7, 0.1); health care (2.86, 0.1); risk (2.86, 0.1); health (2.56, 0.5); mental health (2.43, 0.5); internet (1.82, 0.5); ehealth (1.65, 0.5); mhealth (1.58, 0.5); telemedicine (1.46, 0.5); medical informatics (1.34, 0.5); telehealth (1.17, 0.5); systematic review (1.06, 0.5); technology (1.02, 0.5); physical activity (0.95, 0.5); self-management (0.95, 0.5); health literacy (0.92, 0.5); smartphone (0.77, 0.5); anxiety (0.63, 0.5); cancer (0.59, 0.5); adherence (0.56, 0.5); communication (0.56, 0.5); implementation (0.56, 0.5); qualitative research (0.52, 0.5); cognitive behavioral therapy (0.49, 0.5); ehealth literacy (0.45, 1.0); review (0.45, 1.0); mobile apps (0.42, 1.0); hiv (0.42, 1.0); mobile health (0.41, 1.0); education (0.38, 1.0); health information (0.38, 1.0); adolescents (0.38, 1.0); chronic illness (0.38, 1.0); digital divide (0.38, 1.0); validation (0.38, 1.0); literacy (0.38, 1.0); chronic disease (0.34, 1.0); psychosis (0.34, 1.0); older adults (0.34, 1.0); obesity (0.34, 1.0); mobile phone (0.31, 1.0); consumer health information (0.31, 1.0); oncology (0.31, 1.0); engagement (0.31, 1.0); exercise (0.31, 1.0); health behavior (0.31, 1.0); prevention (0.31, 1.0); e-mental health (0.31, 1.0); health promotion (0.27, 1.0); usage (0.27, 1.0); quality of life (0.27, 1.0); social media (0.27, 1.0); noncommunicable diseases (0.27, 1.0); evaluation (0.27, 1.0); mobile applications (0.27, 1.0); behavior change (0.27, 1.0); older adult (0.27, 1.0); adolescent (0.27, 1.0); cancer survivors (0.24, 1.0); motivation (0.24, 1.0); attrition (0.24, 1.0); diabetes (0.24, 1.0); schizophrenia (0.24, 1.0); text messaging (0.24, 1.0); eheals (0.24, 1.0); survey (0.24, 1.0) |
| **#14 self-management** | 5 | 2008 | self-management; kidney transplantation; life; physical activity; quality; colorectal cancer survivors; computer literacy; cancer survivorship; breast cancer survivors; health literacy \| systematic review; research; multidisciplinary approach; remote monitoring; telemedicine; development; implementation; cardiovascular diseases; mobile apps; chronic kidney disease | self-management (30.45, 1.0E-4); rare disease (7.71, 0.01); mixed methods (7.71, 0.01); computer literacy (7.71, 0.01); access to health care (7.71, 0.01); chronic care model (7.71, 0.01); spinal cord injury (7.71, 0.01); kidney transplantation (7.71, 0.01); chronic illness management (7.71, 0.01); systemic sclerosis (7.71, 0.01); cancer survivorship (4.98, 0.05); migraine (4.98, 0.05); self-care (4.98, 0.05); colorectal cancer survivors (4.98, 0.05); prostate cancer survivors (4.98, 0.05); or hip (3.98, 0.05); research and development (3.98, 0.05); knee and (3.98, 0.05); osteoarthritis (3.98, 0.05); best practices (3.98, 0.05); behavioural change techniques (3.98, 0.05); implementation science (3.98, 0.05); persuasive design (3.98, 0.05); oa (3.98, 0.05); remote monitoring (3.98, 0.05); breast cancer survivors (3.34, 0.1); chronic kidney disease (3.34, 0.1); multidisciplinary approach (3.34, 0.1); nursing (3.34, 0.1); patient participation (3.34, 0.1); meta-ethnography (3.34, 0.1); mental health (2.94, 0.1); machine learning (2.23, 0.5); cardiovascular diseases (2.23, 0.5); quality of life (1.98, 0.5); social media (1.98, 0.5); evaluation (1.98, 0.5); systematic review (1.94, 0.5); depression (1.85, 0.5); oncology (1.78, 0.5); exercise (1.78, 0.5); chronic disease (1.6, 0.5); education (1.44, 0.5); telehealth (1.41, 0.5); digital health (1.37, 0.5); ehealth (1.36, 0.5); mobile apps (1.3, 0.5); hiv (1.3, 0.5); technology (1.24, 0.5); mobile health (1.19, 0.5); social support (1.06, 0.5); qualitative research (0.96, 0.5); smartphone (0.94, 0.5); implementation (0.87, 0.5); primary care (0.85, 0.5); cancer (0.79, 0.5); anxiety (0.76, 0.5); adherence (0.68, 0.5); communication (0.68, 0.5); mhealth (0.67, 0.5); medical informatics (0.59, 0.5); cognitive behavioral therapy (0.59, 0.5); ehealth literacy (0.55, 0.5); review (0.55, 0.5); health information (0.46, 0.5); adolescents (0.46, 0.5); chronic illness (0.46, 0.5); digital divide (0.46, 0.5); validation (0.46, 0.5); literacy (0.46, 0.5); psychosis (0.42, 1.0); older adults (0.42, 1.0); obesity (0.42, 1.0); mobile phone (0.37, 1.0); consumer health information (0.37, 1.0); engagement (0.37, 1.0); health behavior (0.37, 1.0); prevention (0.37, 1.0); e-mental health (0.37, 1.0); health promotion (0.33, 1.0); usage (0.33, 1.0); noncommunicable diseases (0.33, 1.0); mobile applications (0.33, 1.0); behavior change (0.33, 1.0); older adult (0.33, 1.0); adolescent (0.33, 1.0); health literacy (0.3, 1.0); cancer survivors (0.29, 1.0); motivation (0.29, 1.0); attrition (0.29, 1.0); diabetes (0.29, 1.0); schizophrenia (0.29, 1.0); text messaging (0.29, 1.0); eheals (0.29, 1.0); health (0.29, 1.0); survey (0.29, 1.0); copd (0.29, 1.0); intervention (0.29, 1.0); physical activity (0.27, 1.0); psychiatry (0.24, 1.0) |
| **#15 ehealth** | 5 | 2013 | telemedicine; digital health; hypertension prevention; medical technology; mental health apps; smartphones; mobile mental health; ehealth; emotions; multimedia \| mental health; user engagement; health information portal; smartphone; accessibility; telehealth technology; technology enablers; frontline care; everyday technology; e-mental health | 0 |
