# Supplementary material for: Analysis of E-mental health research: mapping the relationship between information technology and mental healthcare
Source: BMC Psychiatry. 2022 Jan 25;22:57. doi: 10.1186/s12888-022-03713-9 (PMC8787445; doi:10.1186/s12888-022-03713-9)
Supplement: Supplementary file 2 — Additional file 2. [file 12888_2022_3713_MOESM2_ESM.docx]

**Appendix 2.**

The top 20 diseases with the IT used in e-mental health (283 entity names)

| Top 20 Diseases | Information Technology | Weight degree |
| --- | --- | --- |
|  |  |  |
| 1. Cancer 2. Diabetes 3. Blood pressure 4. Smoking cessation 5. Dementia 6. Stroke 7. Hypertension 8. Schizophrenia 9. Obesity 10. Breast cancer 11. Asthma 12. Psychosis 13. Bipolar 14. Cardiovascular disease 15. Heart failure 16. Chronic pain 17. Type 2 diabetes 18. Anxiety disorders 19. Arthritis 20. Insomnia | online | 2478 |
|  | measures | 2365 |
|  | mobile | 2358 |
|  | content | 2242 |
|  | video | 2160 |
|  | interactive | 2065 |
|  | treatment as usual | 1996 |
|  | telephone | 1946 |
|  | mobile phone | 1905 |
|  | measure | 1866 |
|  | phone | 1852 |
|  | protocol | 1807 |
|  | email | 1630 |
|  | smartphone | 1562 |
|  | website | 1562 |
|  | mobile health | 1459 |
|  | remote | 1400 |
|  | informed consent | 1399 |
|  | randomized controlled trial | 1391 |
|  | internet | 1376 |
|  | text messaging | 1336 |
|  | instructions | 1293 |
|  | text message | 1241 |
|  | message | 1237 |
|  | monitor | 1221 |
|  | disease management | 1197 |
|  | virtual | 1192 |
|  | search | 1143 |
|  | telehealth | 1138 |
|  | security | 1134 |
|  | games | 1126 |
|  | android | 1095 |
|  | collaboration | 1076 |
|  | sites | 1047 |
|  | network | 1016 |
|  | sensor | 1009 |
|  | hivaids | 1005 |
|  | videoconferencing | 982 |
|  | clinical trials | 975 |
|  | social media | 971 |
|  | body mass index | 945 |
|  | tablet | 940 |
|  | utility | 916 |
|  | clients | 893 |
|  | randomised controlled trial | 854 |
|  | media | 845 |
|  | cognitive behavioral therapy | 840 |
|  | facebook | 835 |
|  | database | 833 |
|  | image | 831 |
|  | audio | 810 |
|  | letter | 808 |
|  | medical records | 808 |
|  | transmission | 804 |
|  | general practitioner | 799 |
|  | connection | 798 |
|  | transition | 796 |
|  | navigation | 783 |
|  | ehealth | 779 |
|  | social | 771 |
|  | distance | 769 |
|  | mobile phones | 743 |
|  | general practice | 734 |
|  | randomized controlled trials | 724 |
|  | algorithm | 718 |
|  | mobile devices | 711 |
|  | heart rate | 710 |
|  | cardiac rehabilitation | 709 |
|  | interface | 684 |
|  | wireless | 674 |
|  | short message service | 670 |
|  | mobile technology | 669 |
|  | calculator | 665 |
|  | remote monitoring | 652 |
|  | iphone | 634 |
|  | real-time | 626 |
|  | simulation | 621 |
|  | confidentiality | 613 |
|  | electronic health record | 592 |
|  | case managers | 587 |
|  | social networks | 580 |
|  | private | 572 |
|  | server | 570 |
|  | interactive voice response | 569 |
|  | multidisciplinary team | 559 |
|  | apple | 556 |
|  | shared decision making | 553 |
|  | confirmation | 547 |
|  | library | 540 |
|  | diagnostics | 539 |
|  | user satisfaction | 536 |
|  | notification | 536 |
|  | mhealth | 534 |
|  | static | 534 |
|  | odds ratio | 533 |
|  | decision support | 530 |
|  | prototype | 530 |
|  | clinical practice guidelines | 521 |
|  | veterans affairs | 518 |
|  | general practitioners | 511 |
|  | mobile technologies | 505 |
|  | emergency room | 502 |
|  | social network | 499 |
|  | health records | 494 |
|  | music | 491 |
|  | electronic medical record | 487 |
|  | randomised controlled trials | 486 |
|  | ecological momentary assessment | 486 |
|  | family planning | 486 |
|  | behavior change techniques | 484 |
|  | camera | 484 |
|  | activities of daily living | 483 |
|  | health management | 482 |
|  | composite | 480 |
|  | system usability scale | 479 |
|  | cognitive behavior therapy | 478 |
|  | nursing home | 473 |
|  | electronic health records | 470 |
|  | decision support system | 465 |
|  | level of satisfaction | 458 |
|  | twitter | 455 |
|  | resolution | 455 |
|  | swipe | 453 |
|  | multimedia | 445 |
|  | photo | 441 |
|  | user interface | 441 |
|  | internal medicine | 440 |
|  | information security | 439 |
|  | alert | 439 |
|  | channel | 433 |
|  | primary care physician | 430 |
|  | analog | 426 |
|  | booklet | 422 |
|  | total cholesterol | 421 |
|  | operation | 420 |
|  | cell phone | 418 |
|  | service quality | 417 |
|  | social networking | 410 |
|  | primary care physicians | 408 |
|  | the communication | 405 |
|  | blood glucose monitoring | 401 |
|  | telephone support | 397 |
|  | range of motion | 394 |
|  | electronic medical records | 394 |
|  | community health worker | 387 |
|  | television | 382 |
|  | communication technology | 372 |
|  | higher education | 372 |
|  | alerts | 369 |
|  | body area network | 368 |
|  | sf-36 | 368 |
|  | antenatal care | 367 |
|  | anova | 367 |
|  | notice | 361 |
|  | nurse practitioner | 359 |
|  | end user | 358 |
|  | label | 355 |
|  | query | 354 |
|  | second life | 354 |
|  | calendar | 349 |
|  | central register of controlled trials | 343 |
|  | virtual world | 341 |
|  | virtual reality | 339 |
|  | limited english proficiency | 338 |
|  | optimisation | 337 |
|  | health information technology | 336 |
|  | dashboard | 335 |
|  | operating system | 334 |
|  | noise | 334 |
|  | prompt | 334 |
|  | google play | 331 |
|  | chronic disease management | 331 |
|  | information and communication technology | 330 |
|  | care managers | 329 |
|  | personal health records | 326 |
|  | driver | 323 |
|  | setup | 323 |
|  | segment | 323 |
|  | patient-reported outcomes | 322 |
|  | educational program | 322 |
|  | patient portal | 320 |
|  | touch screen | 314 |
|  | clinical decision support system | 313 |
|  | medical devices | 313 |
|  | hrqol | 312 |
|  | conferencing | 303 |
|  | peak expiratory flow | 302 |
|  | evidence-based practice | 295 |
|  | cascade | 295 |
|  | authentication | 291 |
|  | person-centered care | 290 |
|  | patient-centered care | 290 |
|  | peripheral | 290 |
|  | optimization | 286 |
|  | information and communication technologies | 285 |
|  | knowledge base | 284 |
|  | adverse event | 284 |
|  | information systems | 284 |
|  | blackberry | 279 |
|  | communication technologies | 278 |
|  | parity | 278 |
|  | ehealth literacy scale | 277 |
|  | timeline | 275 |
|  | registered nurses | 274 |
|  | technology assessment | 274 |
|  | technical support | 274 |
|  | tofhla | 273 |
|  | p-value | 273 |
|  | machine learning | 273 |
|  | behavior change communication | 271 |
|  | update | 271 |
|  | health informatics | 269 |
|  | clinical decision support | 269 |
|  | hints | 269 |
|  | patient-provider communication | 266 |
|  | multidisciplinary teams | 266 |
|  | least squares | 265 |
|  | output | 265 |
|  | interquartile range | 262 |
|  | download | 262 |
|  | health information systems | 262 |
|  | personal health record | 261 |
|  | working group | 261 |
|  | low vision | 259 |
|  | embedded | 259 |
|  | occupational therapists | 255 |
|  | information exchange | 255 |
|  | shared decision-making | 252 |
|  | complementary and alternative medicine | 251 |
|  | participatory design | 247 |
|  | healthcare professional | 247 |
|  | window | 246 |
|  | personal digital assistant | 244 |
|  | healthcare provider | 243 |
|  | developer | 243 |
|  | academic performance | 242 |
|  | analysis of variance | 241 |
|  | analogue | 241 |
|  | array | 238 |
|  | texting | 238 |
|  | tablet computer | 235 |
|  | central nervous system | 233 |
|  | interrupted time series | 230 |
|  | capture | 230 |
|  | electronic prescribing | 226 |
|  | computerized decision support | 226 |
|  | extract | 226 |
|  | open access | 225 |
|  | computer-based assessment | 224 |
|  | central server | 223 |
|  | cognitive rehabilitation | 223 |
|  | home telemonitoring | 220 |
|  | study group | 220 |
|  | modulation | 218 |
|  | housekeeping | 216 |
|  | socio-economic status | 216 |
|  | personal health management | 215 |
|  | comprehensive health enhancement support system | 214 |
|  | sound | 212 |
|  | emerging technologies | 211 |
|  | digital divide | 210 |
|  | personal health information | 210 |
|  | interactive multimedia | 209 |
|  | programming | 208 |
|  | disease management programs | 207 |
|  | health service delivery | 206 |
|  | quality indicator | 206 |
|  | e-mail | 204 |
|  | high frequency | 204 |
|  | heart rate variability | 202 |
|  | desktop | 201 |
|  | personal computer | 201 |
|  | data mining | 197 |
|  | principal investigator | 197 |
|  | itunes | 193 |
|  | bulletin board | 193 |
|  | long-term care | 187 |
|  | odds ratios | 184 |
|  | clinical information | 178 |
|  | mental health professionals | 172 |
|  | global positioning system | 172 |
|  | computer use | 170 |
|  | attention control | 166 |
