# Supplementary material for: Analysis of E-mental health research: mapping the relationship between information technology and mental healthcare
Source: BMC Psychiatry. 2022 Jan 25;22:57. doi: 10.1186/s12888-022-03713-9 (PMC8787445; doi:10.1186/s12888-022-03713-9)
Supplement: Supplementary file 3 — Additional file 3. [file 12888_2022_3713_MOESM3_ESM.docx]

**Appendix 3**

Top 20 Disease entities and relationships in e-mental health

| **Cancer** | | | | | | |
| --- | --- | --- | --- | --- | --- | --- |
| **Symptoms** | Weighted Degree | **Treatment** | Weighted Degree | **IT** | Weighted Degree | |
| depression | 99 | screening | 80 | online | 68 | |
| fatigue | 79 | supportive care | 37 | mobile | 57 | |
| insight | 7 | surgery | 27 | content | 53 | |
| insulin | 5 | discussion | 25 | telehealth | 37 | |
| discharge | 3 | chemotherapy | 22 | video | 34 | |
|  |  | psychosocial assessment | 20 | mobile phone | 26 | |
|  |  | empowerment | 19 | telephone | 25 | |
|  |  | acupuncture | 16 | collaboration | 24 | |
|  |  | advice | 14 | measures | 22 | |
|  |  | adjustment | 14 | search | 22 | |
|  |  | mammogram | 14 | interactive | 21 | |
|  |  | pain management | 13 | phone | 20 | |
|  |  | symptom management | 12 | website | 20 | |
|  |  | radiation | 12 | measure | 19 | |
|  |  | consultation | 11 | mobile devices | 19 | |
|  |  | clinical trial | 11 | mobile health | 17 | |
|  |  | measurement | 10 | optimisation | 16 | |
|  |  | counseling | 7 | message | 15 | |
|  |  | prescription | 6 | clinical trials | 14 | |
|  |  | radiation therapy | 6 | email | 14 | |
|  |  | patient education | 6 | image | 14 | |
|  |  | meetings | 6 | internet | 14 | |
|  |  | biopsy | 6 | protocol | 14 | |
|  |  | examination | 6 | database | 13 | |
|  |  | sigmoidoscopy | 6 | diagnostics | 12 | |
|  |  | barium enema | 6 | remote | 12 | |
|  |  | mammography | 6 | smartphone | 12 | |
|  |  | stimulation | 5 | virtual | 11 | |
|  |  | pain relief | 5 | primary care physician | 11 | |
|  |  | risk assessment | 4 | media | 10 | |
| **Diabetes** | | | | | | |
| **Symptoms** | Weighted Degree | **Treatment** | Weighted Degree | **IT** | Weighted Degree | |
| depression | 219 | screening | 54 | measures | 68 | |
| insulin | 93 | counseling | 30 | mobile phone | 61 | |
| discharge | 10 | advice | 25 | mobile | 41 | |
| fatigue | 6 | surgery | 25 | content | 38 | |
| insight | 2 | empowerment | 23 | telephone | 38 | |
| epidural | 2 | measurement | 17 | text messaging | 36 | |
| burning | 2 | discussion | 16 | measure | 32 | |
| sharp | 2 | hospital admission | 14 | smartphone | 29 | |
| excretion | 2 | case management | 13 | online | 27 | |
| eye problems | 2 | adjustment | 12 | telehealth | 27 | |
| phobia | 2 | consultation | 11 | body mass index | 25 | |
|  |  | prescription | 10 | text message | 24 | |
|  |  | patient education | 9 | remote | 21 | |
|  |  | adjustments | 8 | disease management | 19 | |
|  |  | viral load | 8 | video | 19 | |
|  |  | observation | 7 | interactive | 18 | |
|  |  | scheduling | 7 | mobile health | 17 | |
|  |  | blood test | 6 | message | 16 | |
|  |  | immunization | 5 | phone | 15 | |
|  |  | secondary prevention | 5 | protocol | 14 | |
|  |  | clinical trial | 5 | hivaids | 13 | |
|  |  | aspiration | 5 | insulin therapy | 12 | |
|  |  | physical therapy | 4 | website | 12 | |
|  |  | expression | 4 | glucose tolerance test | 12 | |
|  |  | glucose measurement | 4 | total cholesterol | 12 | |
|  |  | injection | 4 | blood glucose monitoring | 11 | |
|  |  | examination | 4 | primary care physician | 11 | |
|  |  | opportunistic screening | 4 | search | 11 | |
|  |  | influenza vaccination | 4 | collaboration | 10 | |
|  |  | acupuncture | 4 | community health workers | 10 | |
| **Blood pressure** | | | | | | |
| **Symptoms** | Weighted Degree | **Treatment** | Weighted Degree | **IT** | Weighted Degree | |
| depression | 7 | measurement | 26 | heart rate | 46 | |
| insulin | 7 | screening | 15 | measures | 45 | |
| excretion | 6 | counseling | 13 | body mass index | 37 | |
| fatigue | 5 | advice | 11 | mobile phone | 31 | |
| discharge | 5 | prescription | 7 | wireless | 25 | |
| insight | 3 | observation | 6 | mobile | 23 | |
| constant | 3 | surgery | 6 | measure | 17 | |
| suicidal thoughts | 2 | physical examination | 5 | monitor | 17 | |
| reflux | 2 | discussion | 5 | online | 13 | |
| intermittent | 2 | empowerment | 4 | diastolic blood pressure | 13 | |
| migraine | 1 | pain management | 4 | email | 11 | |
| judgement | 1 | meetings | 4 | smartphone | 11 | |
|  |  | elevation | 4 | telephone | 11 | |
|  |  | patient education | 3 | text messaging | 10 | |
|  |  | consultation | 3 | total cholesterol | 10 | |
|  |  | stroke prevention | 3 | content | 9 | |
|  |  | radiation | 3 | c-reactive protein | 9 | |
|  |  | examination | 3 | interquartile range | 9 | |
|  |  | stress management | 2 | phone | 8 | |
|  |  | pulse oximetry | 2 | systolic blood pressure | 8 | |
|  |  | laboratory studies | 2 | medical devices | 8 | |
|  |  | immunization | 2 | interactive | 7 | |
|  |  | viral load | 2 | message | 7 | |
|  |  | hospital admission | 2 | protocol | 7 | |
|  |  | spirometry | 2 | remote | 6 | |
|  |  | risk management | 2 | tablet | 6 | |
|  |  | injection | 2 | virus | 6 | |
|  |  | removal | 2 | randomised controlled trial | 6 | |
|  |  | scheduling | 2 | home monitoring | 5 | |
|  |  | influenza vaccination | 2 | instructions | 5 | |
| **Smoking cessation** | | | | | | |
| **Symptoms** | Weighted Degree | **Treatment** | Weighted Degree | **IT** | Weighted Degree | |
| depression | 37 | counseling | 47 | mobile | 54 | |
| discharge | 5 | advice | 17 | mobile phone | 52 | |
| chest pain | 4 | discussion | 16 | text messaging | 51 | |
| epidural | 2 | stress management | 10 | text message | 44 | |
| fatigue | 2 | screening | 7 | content | 37 | |
| migraine | 2 | pain management | 7 | smartphone | 21 | |
| irritable | 2 | patient education | 6 | online | 19 | |
| insight | 1 | relapse prevention | 5 | measures | 16 | |
| insulin | 1 | suicide prevention | 4 | interactive | 15 | |
| dysplasia | 1 | radiation | 3 | telephone | 14 | |
| major depressive episode | 1 | immunization | 3 | email | 12 | |
| depressive episodes | 1 | symptom management | 3 | message | 12 | |
| phobia | 1 | consultation | 2 | measure | 12 | |
|  |  | chronic medical | 2 | randomized controlled trial | 11 | |
|  |  | adjustment | 2 | mobile health | 11 | |
|  |  | measurement | 2 | phone | 10 | |
|  |  | mood management | 2 | set a | 9 | |
|  |  | notifications | 2 | video | 9 | |
|  |  | hospital admission | 2 | mobile phones | 7 | |
|  |  | stroke prevention | 2 | randomized controlled trials | 7 | |
|  |  | examination | 2 | clinical practice guidelines | 6 | |
|  |  | laboratory studies | 2 | breath | 6 | |
|  |  | ileostomy | 2 | cardiac rehabilitation | 6 | |
|  |  | acupuncture | 2 | social network | 6 | |
|  |  | empowerment | 2 | android | 5 | |
|  |  | bypass graft | 2 | behavior change techniques | 5 | |
|  |  | heat therapy | 2 | facebook | 5 | |
|  |  | home visit | 2 | media | 5 | |
|  |  | relaxation therapy | 2 | patch | 5 | |
|  |  | telephone follow-up | 2 |  |  | |
| **Dementia** | | | | | | |
| **Symptoms** | Weighted Degree | **Treatment** | Weighted Degree | **IT** | Weighted Degree | |
| depression | 24 | screening | 7 | mobile | 25 | |
| discharge | 2 | counseling | 6 | big data | 16 | |
| migraine | 2 | surgery | 6 | tablet | 12 | |
| suicidal thoughts | 2 | adjustment | 5 | cognitive rehabilitation | 12 | |
| orientation | 2 | stimulation | 4 | remote monitoring | 11 | |
| insulin | 1 | chemotherapy | 4 | measures | 9 | |
| fatigue | 1 | measurement | 3 | videoconferencing | 9 | |
| drawing | 1 | discussion | 3 | assistive technology | 7 | |
| pressing | 1 | advice | 3 | content | 7 | |
| suicide attempt | 1 | psychoeducation | 2 | robot | 7 | |
|  |  | case management | 2 | nursing home | 7 | |
|  |  | secondary prevention | 2 | measure | 6 | |
|  |  | pain assessment | 2 | navigation | 6 | |
|  |  | empowerment | 2 | decision support | 6 | |
|  |  | elevation | 2 | smartphone | 6 | |
|  |  | occupational therapy | 2 | search | 6 | |
|  |  | relaxation therapy | 2 | mobile devices | 6 | |
|  |  | telephone follow-up | 2 | hrqol | 6 | |
|  |  | heart transplant | 2 | android | 5 | |
|  |  | safety education | 2 | health and safety | 5 | |
|  |  | fixation | 2 | informed consent | 5 | |
|  |  | prescription | 2 | mobile phone | 5 | |
|  |  | patient education | 2 | protocol | 5 | |
|  |  | perfusion | 2 | e-learning | 5 | |
|  |  | risk management | 2 | story | 5 | |
|  |  | creatinine measurement | 1 | games | 4 | |
|  |  | examination | 1 | calendar | 4 | |
|  |  | notifications | 1 | clients | 4 | |
|  |  | nursing care | 1 | composite | 4 | |
|  |  | mobilization | 1 | mobile technology | 4 | |
| **Stroke** | | | | | | |
| **Symptoms** | Weighted Degree | **Treatment** | Weighted Degree | **IT** | Weighted Degree | |
| depression | 25 | screening | 19 | games | 13 | |
| discharge | 21 | secondary prevention | 14 | video | 12 | |
| fatigue | 7 | stroke prevention | 8 | measures | 11 | |
| insulin | 5 | primary prevention | 8 | mobile | 10 | |
| phobia | 2 | surgery | 6 | attack | 7 | |
| hyperactivity | 1 | discussion | 5 | body mass index | 7 | |
| migraine | 1 | chronic medical | 4 | website | 7 | |
| chest pain | 1 | counseling | 4 | message | 6 | |
| constant | 1 | skills training | 4 | email | 6 | |
|  |  | heart transplant | 3 | text message | 6 | |
|  |  | examination | 3 | activities of daily living | 5 | |
|  |  | measurement | 3 | tablet | 5 | |
|  |  | health assessment | 3 | android | 4 | |
|  |  | notifications | 3 | diagnostics | 4 | |
|  |  | echocardiography | 3 | mechanical | 4 | |
|  |  | closure | 3 | mobile health | 4 | |
|  |  | meetings | 3 | content | 4 | |
|  |  | advice | 2 | virtual | 4 | |
|  |  | carotid endarterectomy | 2 | scanner | 4 | |
|  |  | follow-up visit | 2 | smartphone | 4 | |
|  |  | clinical trial | 2 | disease management | 3 | |
|  |  | opportunistic screening | 2 | interactive | 3 | |
|  |  | immunization | 2 | letter | 3 | |
|  |  | injection | 2 | monitor | 3 | |
|  |  | prescription | 2 | phone | 3 | |
|  |  | removal | 2 | severe mental illness | 3 | |
|  |  | scheduling | 2 | telephone | 3 | |
|  |  | influenza vaccination | 2 | notification | 3 | |
|  |  | medical service | 2 | protocol | 3 | |
|  |  | risk management | 2 | sciences | 3 | |
| **Hypertension** | | | | | | |
| **Symptoms** | Weighted Degree | **Treatment** | Weighted Degree | **IT** | Weighted Degree | |
| depression | 45 | screening | 32 | online | 24 | |
| insulin | 5 | discussion | 8 | body mass index | 18 | |
| polydipsia | 3 | advice | 7 | measures | 17 | |
| discharge | 3 | surgery | 7 | mobile | 12 | |
| migraine | 2 | consultation | 6 | diastolic blood pressure | 11 | |
| epidural | 2 | pain management | 6 | family planning | 9 | |
| fatigue | 2 | chronic medical | 6 | mobile health | 8 | |
| burning | 2 | prescription | 5 | smartphone | 8 | |
| sharp | 2 | empowerment | 4 | total cholesterol | 8 | |
| hyperactivity | 1 | case management | 4 | wireless | 8 | |
| intermittent | 1 | viral load | 4 | mobile phone | 7 | |
| insight | 1 | primary prevention | 4 | telephone | 7 | |
| reflux | 1 | influenza vaccination | 4 | decision support | 6 | |
|  |  | skills training | 4 | hivaids | 6 | |
|  |  | medication review | 3 | middle-income countries | 6 | |
|  |  | immunization | 3 | phone | 5 | |
|  |  | opportunistic screening | 3 | randomized controlled trial | 5 | |
|  |  | physical therapy | 3 | remote | 5 | |
|  |  | counseling | 3 | chronic disease management | 5 | |
|  |  | notifications | 2 | text message | 5 | |
|  |  | triage | 2 | text messaging | 5 | |
|  |  | hospital admission | 2 | output | 5 | |
|  |  | hemodialysis | 2 | content | 4 | |
|  |  | transplantation | 2 | authentication | 4 | |
|  |  | clinical trial | 2 | interactive | 4 | |
|  |  | patient education | 2 | internet | 4 | |
|  |  | measurement | 2 | clinical trials | 4 | |
|  |  | tetanus vaccination | 2 | people living with hiv | 4 | |
|  |  | injection | 2 | vaccine | 4 | |
|  |  | removal | 2 | minas gerais | 4 | |
| **Schizophrenia** | | | | | | |
| **Symptoms** | Weighted Degree | **Treatment** | Weighted Degree | **IT** | Weighted Degree | |
| depression | 45 | psychoeducation | 4 | smartphone | 57 | |
| delusion | 4 | chemotherapy | 4 | mobile | 25 | |
| discharge | 2 | skills training | 4 | measures | 24 | |
| migraine | 2 | reinforcement | 4 | online | 20 | |
| hyperactivity | 2 | chronic medical | 3 | phone | 16 | |
| suicidal thoughts | 2 | prescription | 3 | mobile phone | 13 | |
| insulin | 1 | screening | 3 | text messaging | 10 | |
| insight | 1 | relapse prevention | 2 | serious mental illness | 10 | |
| apathy | 1 | computed tomography | 2 | texting | 9 | |
| bronchial asthma | 1 | revision | 2 | content | 8 | |
|  |  | stress management | 2 | text message | 8 | |
|  |  | discussion | 2 | utility | 7 | |
|  |  | first aid | 2 | internet | 6 | |
|  |  | adjustments | 2 | mobile health | 6 | |
|  |  | circumcision | 2 | mobile technologies | 6 | |
|  |  | influenza vaccination | 2 | social media | 6 | |
|  |  | liver transplant | 2 | clients | 5 | |
|  |  | physical therapy | 2 | email | 5 | |
|  |  | mammography | 2 | video | 5 | |
|  |  | breastfeeding education | 2 | measure | 5 | |
|  |  | symptom management | 2 | telephone | 5 | |
|  |  | measurement | 2 | search | 5 | |
|  |  | primary prevention | 2 | alert | 4 | |
|  |  | examination | 2 | anger | 4 | |
|  |  | empowerment | 1 | games | 4 | |
|  |  | risk management | 1 | geolocation | 4 | |
|  |  | expression | 1 | health belief model | 4 | |
|  |  | health assessment | 1 | mobile devices | 4 | |
|  |  | vocational training | 1 | virtual environments | 4 | |
|  |  | surgery | 1 | letter | 3 | |
| **Obesity** | | | | | | |
| **Symptoms** | Weighted Degree | **Treatment** | Weighted Degree | **IT** | Weighted Degree | |
| depression | 32 | discussion | 7 | mobile | 14 | |
| insulin | 5 | measurement | 7 | measures | 13 | |
| migraine | 4 | counseling | 6 | protocol | 12 | |
| insight | 4 | empowerment | 6 | body mass index | 10 | |
| irrelevant | 4 | clinical trial | 6 | smartphone | 8 | |
| polydipsia | 2 | screening | 6 | telephone | 7 | |
| excretion | 2 | surgery | 5 | online | 5 | |
| discharge | 2 | hemodialysis | 4 | video | 5 | |
|  |  | anticipatory guidance | 4 | interactive | 5 | |
|  |  | consultation | 3 | arterial hypertension | 4 | |
|  |  | primary prevention | 3 | health management | 4 | |
|  |  | secondary prevention | 3 | human immunodeficiency virus | 4 | |
|  |  | adjustment | 3 | blood glucose monitoring | 4 | |
|  |  | observation | 3 | measure | 4 | |
|  |  | prescription | 2 | clinical trials | 4 | |
|  |  | advice | 2 | telehealth | 4 | |
|  |  | pain assessment | 2 | social networks | 4 | |
|  |  | laboratory studies | 2 | message | 4 | |
|  |  | pain management | 2 | minas gerais | 4 | |
|  |  | amputation | 2 | mhealth | 4 | |
|  |  | aspiration | 2 | mobile health | 4 | |
|  |  | expression | 2 | developmental disabilities | 4 | |
|  |  | perfusion | 2 | trunk | 4 | |
|  |  | endocrinology service | 2 | network | 4 | |
|  |  | glucose measurement | 2 | body fat | 3 | |
|  |  | hospital admission | 2 | noncommunicable diseases | 3 | |
|  |  | intramuscular injection | 2 | nursing home | 3 | |
|  |  | liver transplant | 2 | remote | 3 | |
|  |  | medical assessment | 2 | remote monitoring | 3 | |
|  |  | patient education | 2 |  |  | |
| **Breast cancer** | | | | | | |
| **Symptoms** | Weighted Degree | **Treatment** | Weighted Degree | **IT** | Weighted Degree | |
| depression | 19 | chemotherapy | 17 | mobile | 27 | |
| fatigue | 15 | surgery | 15 | online | 19 | |
| insulin | 4 | screening | 13 | content | 17 | |
| discharge | 3 | mammography | 12 | telephone | 15 | |
| hyperactivity | 2 | discussion | 9 | text message | 12 | |
| migraine | 2 | supportive care | 9 | mobile phone | 11 | |
| fracture | 2 | measurement | 9 | chess | 9 | |
| insight | 1 | risk assessment | 7 | message | 8 | |
| scared | 1 | symptom management | 7 | measure | 7 | |
| intermittent | 1 | breast self-examination | 6 | interactive | 6 | |
| sharp | 1 | clinical trial | 6 | mobile health | 6 | |
|  |  | adjustment | 5 | network analysis | 6 | |
|  |  | empowerment | 4 | email | 5 | |
|  |  | advice | 4 | internet | 5 | |
|  |  | pain management | 4 | cognitive behavior therapy | 5 | |
|  |  | counseling | 4 | phone | 5 | |
|  |  | scheduling | 4 | search | 5 | |
|  |  | notifications | 3 | website | 5 | |
|  |  | mammogram | 3 | audio | 4 | |
|  |  | examination | 3 | monitor | 4 | |
|  |  | secondary prevention | 3 | facebook | 4 | |
|  |  | removal | 3 | smartphone | 4 | |
|  |  | immunization | 3 | connection | 4 | |
|  |  | consultation | 2 | yahoo | 4 | |
|  |  | prescription | 2 | confidentiality | 3 | |
|  |  | reassuring | 2 | breath | 3 | |
|  |  | radiation therapy | 2 | instructions | 3 | |
|  |  | expression | 2 | meter | 3 | |
|  |  | endocrine therapy | 2 | video | 3 | |
|  |  | influenza vaccination | 2 | measures | 3 | |
| **Asthma** | | | | | | |
| **Symptoms** | Weighted Degree | **Treatment** | Weighted Degree | **IT** | Weighted Degree | |
| depression | 27 | screening | 8 | measures | 14 | |
| fatigue | 5 | chemotherapy | 6 | mobile phone | 14 | |
| discharge | 4 | surgery | 5 | text message | 14 | |
| migraine | 2 | advice | 5 | text messaging | 12 | |
| epidural | 2 | physical therapy | 5 | interactive | 11 | |
| insulin | 1 | immunization | 5 | content | 10 | |
| tightness | 1 | prescription | 4 | message | 10 | |
| coughing | 1 | measurement | 4 | mobile | 10 | |
| insight | 1 | scheduling | 4 | phone | 10 | |
|  |  | patient education | 4 | smartphone | 8 | |
|  |  | consultation | 4 | online | 6 | |
|  |  | discussion | 4 | email | 6 | |
|  |  | adjustment | 3 | telephone | 6 | |
|  |  | symptom management | 2 | mobile health | 5 | |
|  |  | empowerment | 2 | emergency room | 5 | |
|  |  | chronic medical | 2 | antenatal care | 4 | |
|  |  | tetanus vaccination | 2 | chest | 4 | |
|  |  | case management | 2 | disease management | 4 | |
|  |  | injection | 2 | patient-provider communication | 4 | |
|  |  | removal | 2 | randomized controlled trial | 4 | |
|  |  | influenza vaccination | 2 | short message service | 4 | |
|  |  | circumcision | 2 | video | 4 | |
|  |  | liver transplant | 2 | medication errors | 4 | |
|  |  | ileostomy | 2 | middle-income countries | 4 | |
|  |  | suicide prevention | 2 | facebook | 4 | |
|  |  | spirometry | 2 | concept mapping | 4 | |
|  |  | clinical trial | 2 | antiretroviral therapy | 3 | |
|  |  | patient counseling | 2 | mobile technologies | 3 | |
|  |  | blood test | 2 | content analysis | 3 | |
|  |  | intravenous chemotherapy | 1 | mobile phones | 3 | |
| **Psychosis** | | | | | | |
| **Symptoms** | Weighted Degree | **Treatment** | Weighted Degree | **IT** | Weighted Degree | |
| depression | 58 | screening | 6 | online | 44 | |
| discharge | 6 | advice | 5 | mobile phone | 19 | |
| intoxication | 2 | revision | 5 | mobile | 18 | |
| intermittent | 2 | psychoeducation | 4 | clients | 15 | |
| suicide attempt | 2 | discussion | 4 | transition | 14 | |
| depressive episodes | 2 | viral load | 4 | phone | 10 | |
| insulin | 1 | surgery | 3 | smartphone | 10 | |
| irrelevant | 1 | psychological therapies | 3 | email | 9 | |
| insight | 1 | empowerment | 2 | measure | 8 | |
| apathy | 1 | expression | 2 | text message | 8 | |
| hyperactivity | 1 | circumcision | 2 | social media | 6 | |
|  |  | influenza vaccination | 2 | telephone | 6 | |
|  |  | liver transplant | 2 | mobile phones | 5 | |
|  |  | physical therapy | 2 | website | 5 | |
|  |  | self-referral | 2 | interactive | 4 | |
|  |  | obtaining consent | 2 | message | 4 | |
|  |  | counseling | 2 | social network | 4 | |
|  |  | skills training | 2 | measures | 4 | |
|  |  | chemotherapy | 2 | disease management | 4 | |
|  |  | other referral | 2 | update | 4 | |
|  |  | reinforcement | 2 | direct access | 3 | |
|  |  | relapse prevention | 1 | informed consent | 3 | |
|  |  | risk management | 1 | mobile devices | 3 | |
|  |  | heart transplant | 1 | network | 3 | |
|  |  | suicide prevention | 1 | social networking | 3 | |
|  |  | examination | 1 | internet | 3 | |
|  |  | consultation | 1 | video | 3 | |
|  |  | psychosocial assessment | 1 | mobile health | 3 | |
|  |  | prescription | 1 | mobile technology | 3 | |
|  |  | measurement | 1 |  |  | |
| **Bipolar** | | | | | | |
| **Symptoms** | Weighted Degree | **Treatment** | Weighted Degree | **IT** | Weighted Degree | |
| depression | 79 | screening | 7 | smartphone | 28 | |
| depressive episodes | 4 | psychoeducation | 6 | mobile | 26 | |
| hyperactivity | 3 | surgery | 5 | online | 14 | |
| suicidal thoughts | 3 | suicide prevention | 5 | mobile phone | 12 | |
| discharge | 2 | measurement | 4 | streaming | 11 | |
| constant | 2 | advice | 3 | calculator | 7 | |
| necrosis | 2 | observation | 3 | email | 7 | |
| major depressive episode | 2 | physical therapy | 3 | phone | 6 | |
| fatigue | 1 | chemotherapy | 3 | sensor | 6 | |
| migraine | 1 | discussion | 2 | telephone | 6 | |
| suicide attempt | 1 | notifications | 2 | serious mental illness | 6 | |
|  |  | adjustment | 2 | measures | 5 | |
|  |  | clinical trial | 2 | search | 5 | |
|  |  | relapse prevention | 2 | general practitioner | 4 | |
|  |  | circumcision | 2 | mobile health | 4 | |
|  |  | influenza vaccination | 2 | content | 4 | |
|  |  | liver transplant | 2 | early warning systems | 4 | |
|  |  | adjustments | 2 | embedded | 4 | |
|  |  | examination | 2 | hamilton depression rating scale | 4 | |
|  |  | health assessment | 2 | library | 4 | |
|  |  | self-care interventions | 2 | condition monitoring | 4 | |
|  |  | listening | 2 | texting | 4 | |
|  |  | empowerment | 1 | distance | 4 | |
|  |  | consultation | 1 | android | 3 | |
|  |  | prescription | 1 | letter | 3 | |
|  |  | reassuring | 1 | remote | 3 | |
|  |  | pain management | 1 | hivaids | 3 | |
|  |  | lifestyle screening | 1 | gyroscope | 3 | |
|  |  | risk assessment | 1 | instructions | 3 | |
|  |  | supportive care | 1 | internet | 3 | |
| **Cardiovascular disease** | | | | | | |
| **Symptoms** | Weighted Degree | **Treatment** | Weighted Degree | **IT** | Weighted Degree | |
| depression | 23 | secondary prevention | 4 | mobile | 7 | |
| insulin | 1 | screening | 3 | electronic health record | 6 | |
| fatigue | 1 | prescription | 2 | acquired brain injury | 4 | |
|  |  | counseling | 2 | general practitioner | 4 | |
|  |  | search procedure | 2 | andhra pradesh | 3 | |
|  |  | limb amputation | 2 | content | 3 | |
|  |  | peritoneal dialysis | 2 | internet | 3 | |
|  |  | primary prevention | 2 | randomised controlled trial | 3 | |
|  |  | examination | 2 | serious mental illness | 3 | |
|  |  | surgery | 1 | telehealth | 3 | |
|  |  | heart transplant | 1 | adverse event | 2 | |
|  |  | opportunistic screening | 1 | algorithm | 2 | |
|  |  | patient education | 1 | android | 2 | |
|  |  | visual aid | 1 | arterial hypertension | 2 | |
|  |  | community education | 1 | audio | 2 | |
|  |  | chronic medical | 1 | heart rate | 2 | |
|  |  | empowerment | 1 | measure | 2 | |
|  |  | advice | 1 | mobile health | 2 | |
|  |  | dietary advice | 1 | community health worker | 2 | |
|  |  | psychological counseling | 1 | continuing education | 2 | |
|  |  | coronary angiography | 1 | randomised controlled trials | 2 | |
|  |  | immunization | 1 | database | 2 | |
| **Heart failure** | | | | | | |
| **Symptoms** | Weighted Degree | **Treatment** | Weighted Degree | **IT** | Weighted Degree | |
| depression | 16 | screening | 7 | corpora | 12 | |
| discharge | 4 | observation | 7 | phone | 11 | |
| fatigue | 3 | discussion | 6 | smartphone | 11 | |
| insulin | 2 | surgery | 6 | message | 11 | |
| hyperactivity | 1 | advice | 5 | telehealth | 10 | |
|  |  | counseling | 4 | mobile | 7 | |
|  |  | influenza vaccination | 4 | text message | 7 | |
|  |  | measurement | 3 | body mass index | 6 | |
|  |  | prescription | 2 | measures | 6 | |
|  |  | secondary prevention | 2 | mobile phone | 6 | |
|  |  | adjustment | 2 | email | 6 | |
|  |  | immunization | 2 | general practice | 6 | |
|  |  | injection | 2 | hrqol | 6 | |
|  |  | removal | 2 | electronic health record | 6 | |
|  |  | scheduling | 2 | video | 6 | |
|  |  | circumcision | 2 | cardiac rehabilitation | 5 | |
|  |  | liver transplant | 2 | remote | 5 | |
|  |  | physical therapy | 2 | general practitioner | 5 | |
|  |  | cardioversion | 2 | iphone | 5 | |
|  |  | meetings | 2 | private | 5 | |
|  |  | physical examination | 2 | android | 4 | |
|  |  | pulse oximetry | 2 | games | 4 | |
|  |  | case management | 2 | mobile health | 4 | |
|  |  | empowerment | 1 | collaboration | 4 | |
|  |  | consultation | 1 | telephone | 4 | |
|  |  | notifications | 1 | text messaging | 4 | |
|  |  | reassuring | 1 | utility | 4 | |
|  |  | pain management | 1 | heart rate | 4 | |
|  |  | lifestyle screening | 1 | interactive | 4 | |
|  |  | risk assessment | 1 | letter | 4 | |
| **Chronic pain** | | | | | | |
| **Symptoms** | Weighted Degree | **Treatment** | Weighted Degree | **IT** | Weighted Degree | |
| depression | 34 | pain management | 14 | online | 18 | |
| fatigue | 13 | discussion | 10 | telehealth | 18 | |
| insight | 1 | surgery | 5 | content | 16 | |
| suicidal thoughts | 1 | prescription | 4 | mobile phone | 11 | |
| intermittent | 1 | mammogram | 4 | mobile | 9 | |
| neck pain | 1 | opiates | 3 | central nervous system | 6 | |
| aseptic | 1 | acupuncture | 3 | cognitive behavioral therapy | 6 | |
| necrosis | 1 | screening | 3 | music | 6 | |
| irritable | 1 | symptom management | 2 | measures | 5 | |
| knee pain | 1 | stress management | 2 | telephone | 5 | |
| migraine | 1 | heart transplant | 2 | video | 5 | |
|  |  | psychological therapies | 2 | behavior change techniques | 4 | |
|  |  | clinical trial | 2 | conferencing | 4 | |
|  |  | manipulation | 2 | interactive video | 4 | |
|  |  | massage | 2 | array | 4 | |
|  |  | pain relief | 2 | remote | 4 | |
|  |  | pain assessment | 2 | phone | 3 | |
|  |  | adjustment | 2 | socio-economic status | 3 | |
|  |  | preventive treatment | 2 | family medicine | 3 | |
|  |  | counseling | 2 | security | 3 | |
|  |  | laboratory studies | 2 | randomized controlled trial | 3 | |
|  |  | search procedure | 2 | electronic health record | 2 | |
|  |  | revision | 2 | audio | 2 | |
|  |  | chemotherapy | 1 | avatar | 2 | |
|  |  | echocardiography | 1 | informed consent | 2 | |
|  |  | pain rehabilitation | 1 | census | 2 | |
|  |  | consultation | 1 | mobile health | 2 | |
|  |  | observation | 1 | clinical practice guidelines | 2 | |
|  |  | reinforcement | 1 | operation | 2 | |
|  |  | self-care interventions | 1 | cognitive behavior therapy | 2 | |
| **Type 2 diabetes** | | | | | | |
| **Symptoms** | Weighted Degree | **Treatment** | Weighted Degree | **IT** | Weighted Degree | |
| depression | 39 | surgery | 8 | telehealth | 16 | |
| insulin | 24 | screening | 7 | text message | 15 | |
| excretion | 2 | discussion | 7 | mobile phone | 10 | |
| suicidal thoughts | 2 | perfusion | 6 | content | 9 | |
| insight | 1 | counseling | 4 | analogue | 8 | |
| migraine | 1 | advice | 4 | measures | 8 | |
|  |  | aspiration | 4 | mobile | 8 | |
|  |  | endocrinology service | 4 | online | 8 | |
|  |  | glucose measurement | 4 | protocol | 7 | |
|  |  | hospital admission | 4 | remote | 7 | |
|  |  | intramuscular injection | 4 | telephone | 7 | |
|  |  | liver transplant | 4 | body mass index | 6 | |
|  |  | medical assessment | 4 | hba1c | 6 | |
|  |  | empowerment | 3 | total cholesterol | 6 | |
|  |  | adjustment | 3 | informed consent | 5 | |
|  |  | nursing care | 2 | blood glucose monitoring | 5 | |
|  |  | measurement | 2 | randomized controlled trial | 5 | |
|  |  | depression screening | 2 | alice | 4 | |
|  |  | consultation | 2 | internet | 4 | |
|  |  | amputation | 2 | confirmation | 4 | |
|  |  | expression | 2 | randomized controlled trials | 4 | |
|  |  | computed tomography | 2 | health records | 4 | |
|  |  | patient education | 2 | insulin therapy | 4 | |
|  |  | observation | 2 | key in | 4 | |
|  |  | caregiver education | 2 | unesp | 4 | |
|  |  | prescription | 2 | after | 4 | |
|  |  | heart transplant | 1 | instructions | 4 | |
|  |  | adjustments | 1 | minas gerais | 4 | |
|  |  | meetings | 1 | adverse event | 4 | |
|  |  | blood test | 1 | odds ratio | 4 | |
| **Anxiety disorders** | | | | | | |
| **Symptoms** | Weighted Degree | **Treatment** | Weighted Degree | **IT** | Weighted Degree | |
| depression | 50 | psychoeducation | 3 | online | 16 | |
| phobia | 4 | suicide prevention | 2 | mobile | 10 | |
| insight | 2 | revision | 2 | cognitive behavioral therapy | 8 | |
| acrophobia | 2 | evaluating interventions | 2 | email | 7 | |
| insulin | 1 | advice | 2 | anger | 6 | |
| discharge | 1 | psychological therapies | 2 | audio | 6 | |
| irritable | 1 | screening | 2 | measures | 6 | |
| neck pain | 1 | patient education | 2 | internet | 5 | |
| depressive episodes | 1 | chronic medical | 2 | image | 4 | |
| major depressive episode | 1 | surgery | 1 | mobile phone | 4 | |
| fatigue | 1 | heart transplant | 1 | mobile phones | 4 | |
|  |  | measurement | 1 | oasis | 4 | |
|  |  | counseling | 1 | treatment as usual | 4 | |
|  |  | notifications | 1 | telephone | 4 | |
|  |  | health assessment | 1 | video | 4 | |
|  |  | skills training | 1 | android | 3 | |
|  |  | laminectomy | 1 | phone | 3 | |
|  |  | symptom management | 1 | monitor | 3 | |
|  |  | stress management | 1 | randomized controlled trials | 3 | |
|  |  | adjustment | 1 | accessories | 2 | |
|  |  | relapse prevention | 1 | cd-rom | 2 | |
|  |  | psychological assessment | 1 | alert | 2 | |
|  |  |  |  | behavioral patterns | 2 | |
|  |  |  |  | calendar | 2 | |
|  |  |  |  | chat room | 2 | |
|  |  |  |  | sensor | 2 | |
|  |  |  |  | website | 2 | |
|  |  |  |  | spider | 2 | |
|  |  |  |  | clients | 2 | |
|  |  |  |  | cookie | 2 | |
| **Arthritis** | | | | | | |
| **Symptoms** | Weighted Degree | **Treatment** | Weighted Degree | **IT** | Weighted Degree | |
| depression | 15 | discussion | 9 | facebook | 20 | |
| fracture | 2 | advice | 5 | online | 9 | |
| hyperactivity | 1 | immunology | 5 | clinical practice guidelines | 4 | |
| insulin | 1 | observation | 4 | output | 4 | |
| migraine | 1 | chronic medical | 4 | telephone | 4 | |
| chest pain | 1 | prescription | 3 | variant | 4 | |
| mucosa | 1 | empowerment | 2 | interactive | 4 | |
| fatigue | 1 | consultation | 2 | university of technology | 4 | |
| swelling | 1 | amputation | 2 | intention to use | 4 | |
| apathy | 1 | examination | 2 | image | 4 | |
| tender | 1 | synovial biopsy | 2 | evidence-based practice | 4 | |
| necrosis | 1 | injection | 2 | social capital | 4 | |
| dysplasia | 1 | sedation | 2 | electronic medical records | 3 | |
|  |  | notifications | 1 | email | 3 | |
|  |  | reassuring | 1 | phone | 3 | |
|  |  | surgery | 1 | security | 3 | |
|  |  | pain management | 1 | flare | 3 | |
|  |  | lifestyle screening | 1 | internet | 3 | |
|  |  | risk assessment | 1 | measure | 3 | |
|  |  | supportive care | 1 | video | 3 | |
|  |  | symptom management | 1 | perceived ease of use | 3 | |
|  |  | physical therapy | 1 | confidentiality | 2 | |
|  |  | scheduling | 1 | algorithm | 2 | |
|  |  | imaging | 1 | delay | 2 | |
|  |  | physical examination | 1 | behavior change techniques | 2 | |
|  |  | biopsy | 1 | cardiac rehabilitation | 2 | |
|  |  | hemodialysis | 1 | mobile health | 2 | |
|  |  | renal replacement | 1 | clinical practice guideline | 2 | |
|  |  | replacement therapy | 1 | clinical trials | 2 | |
|  |  | ultrasonography | 1 | content | 2 | |
| **Insomnia** | | | | | | |
| **Symptoms** | Weighted Degree | **Treatment** | Weighted Degree | **IT** | Weighted Degree | |
| depression | 74 | discussion | 6 | cognitive behavioral therapy | | 10 |
| fatigue | 12 | relapse prevention | 4 | mobile phone | | 9 |
| apathy | 2 | psychoeducation | 3 | online | | 7 |
| insulin | 1 | stress management | 3 | protocol | | 7 |
| difficulty concentrating | 1 | surgery | 2 | measures | | 5 |
| migraine | 1 | examination | 2 | mobile technologies | | 4 |
| suicidal thoughts | 1 | heart transplant | 1 | website | | 3 |
|  |  | suicide prevention | 1 | measure | | 3 |
|  |  | expression | 1 | anger | | 2 |
|  |  | screening | 1 | beats | | 2 |
|  |  | health assessment | 1 | latency | | 2 |
|  |  | prescription | 1 | clients | | 2 |
|  |  | counseling | 1 | clinical information | | 2 |
|  |  | acupuncture | 1 | facebook | | 2 |
|  |  | measurement | 1 | general internal medicine | | 2 |
|  |  | chemotherapy | 1 | mobile | | 2 |
|  |  | hygiene education | 1 | internet | | 2 |
|  |  | amputation | 1 | modulation | | 2 |
|  |  | revision | 1 | virtual | | 2 |
|  |  | psychological assessment | 1 | noise | | 2 |
|  |  | opiates | 1 | level of satisfaction | | 2 |
|  |  |  |  | breath | 2 | |
|  |  |  |  | remote | 2 | |
|  |  |  |  | sound | 2 | |
|  |  |  |  | story | 2 | |
|  |  |  |  | streaming | 2 | |
|  |  |  |  | analogue | 2 | |
|  |  |  |  | behavioral patterns | 1 | |
|  |  |  |  | perceived value | 1 | |
|  |  |  |  | randomised control trial | 1 | |
